# MicroRNA expression profile and functional analysis reveal that miR-382 is a critical novel gene of alcohol addiction

**DOI:** 10.1002/emmm.201201900

**Published:** 2013-07-22

**Authors:** Jingyuan Li, Jing Li, Xiaojun Liu, Shanshan Qin, Yanzhong Guan, Yuwei Liu, Yunhui Cheng, Xiuwen Chen, Wen Li, Shenming Wang, Ming Xiong, Eldo V Kuzhikandathil, Jiang-Hong Ye, Chunxiang Zhang

**Affiliations:** 1 Department of Pharmacology, Rush University Medical Center, Rush UniversityChicago, IL, USA; 2 Department of Anesthesiology, New Jersey Medical School, University of Medicine and Dentistry of New JerseyNewark, NJ, USA; 3 Department of Pharmacology & Physiology, New Jersey Medical School, University of Medicine and Dentistry of New JerseyNewark, NJ, USA

**Keywords:** alcohol addiction, DeltaFosB, dopamine receptor D1, microRNAs, miR-382

## Abstract

Alcohol addiction is a major social and health concern. Here, we determined the expression profile of microRNAs (miRNAs) in the nucleus accumbens (NAc) of rats treated with alcohol. The results suggest that multiple miRNAs were aberrantly expressed in rat NAc after alcohol injection. Among them, miR-382 was down-regulated in alcohol-treated rats. In both cultured neuronal cells *in vitro* and in the NAc *in vivo*, we identified that the dopamine receptor D1 (*Drd1*) is a direct target gene of miR-382. Via this target gene, miR-382 strongly modulated the expression of DeltaFosB. Moreover, overexpression of miR-382 significantly attenuated alcohol-induced up-regulation of DRD1 and DeltaFosB, decreased voluntary intake of and preference for alcohol and inhibited the DRD1-induced action potential responses. The results indicated that miRNAs are involved in and may represent novel therapeutic targets for alcoholism.

## INTRODUCTION

Alcohol addiction is a major social and health concern (Björk et al, 2010; Ray et al, 2010; Spanagel et al, 2010). Almost 20 million Americans are alcohol dependent or regularly drink alcohol in harmful quantities. It is estimated that about 12% of American adults will experience alcohol (ethanol) addiction at some time in their lives. Because of the limited understanding of the underlying causes of alcohol addiction, effective strategies for treating alcoholism are still lacking (Björk et al, 2010; Ray et al, 2010). There is an urgent need to improve our understanding of the molecular mechanisms of alcohol addiction and to develop novel therapeutic strategies for this complex disorder (Nestler, 2005; Ray et al, 2010).

To date, many studies have been performed to explore the potential signal pathways involved in alcohol intake. Among them, two important signal molecules that are related to alcohol abuse have been identified: dopamine receptor D1 (DRD1) and DeltaFosB. DRD1 is a subtype of the dopamine receptors and is also the most abundant dopamine receptor in the central nervous system. The studies from us and other groups have revealed that the expression and/or activation of DRD1 are significantly increased in the central nervous system in animals after alcohol intake (Lograno et al, 1993; Xiong et al, 2011). Blocking of DRD1 via its antagonist or siRNA is efficient to inhibit the alcohol-induced psychomotor sensitization and addictive behaviour (Abrahao et al, 2011; Bahi and Dreyer, 2012; Czachowski et al, 2012; Hodge et al, 1997). Moreover, knock-out of *Drd1* in mice attenuates alcohol intake as compared to their wild-type littermates (El-Ghundi et al, 1998). DeltaFosB, a truncated splice variant of FosB, is believed to be a critical molecular switch for all drugs of abuse (Nestler et al, 2001; Nestler, 2005). The recent studies from us and other groups have found that the expression of DeltaFosB is significantly up-regulated in animals treated with alcohol (Li et al, 2010; Ozburn et al, 2012; Perrotti et al, 2008; Xiong et al, 2011).

MicroRNAs (miRNAs) have emerged as a novel class of endogenous, small, noncoding RNAs that negatively regulate over 30% of genes in a cell via degradation or translational inhibition of their target mRNAs (Ambros, 2004; Lewis et al, 2005). Functionally, an individual miRNA is important as a transcription factor because it is able to regulate the expression of its multiple target genes (Chen and Rajewsky, 2007). Recent studies have revealed that miRNAs have strong biological functions that may impact almost every aspect of biology and biomedicine (Zhang, 2008). However, the roles of miRNAs in alcohol addiction are still unclear.

To determine the potential roles of miRNAs in alcohol addiction and the potential mechanisms involved, we here use the miRNA microarray to determine the expression profile of miRNAs in the nucleus accumbens (NAc) of brain in a rat model of alcohol intake, and show that multiple miRNAs are aberrantly expressed in rat NAc after alcohol injection. Among them, miR-382 is down-regulated in alcohol-treated rats. We further demonstrate that miR-382 plays an important role in alcohol-seeking behaviour through the DRD1 and DeltaFosB pathway.

## RESULTS

### The expression profile of miRNAs in brain NAc of rats with alcohol intake

Currently, there is no report on the expression signature of miRNAs in NAc of rats treated with alcohol. We thus determined the miRNA expression profiles using miRNA microarray analysis. To perform the experiment, 18 male rats (weighing 150–180 g) were divided into two treatment groups: vehicle (500 µl saline, i.p. bid) or alcohol (1 g/kg, i.p. bid). Seven days later, the animals were sacrificed and their NAc were isolated for miRNA microarray analysis. The results demonstrated, among the 300 detected miRNAs in NAc, multiple miRNAs were aberrantly expressed after treatment with alcohol. MiRNAs that were highly expressed in NAc and over 30% changes in their expression after alcohol treatment and their *t*-test *p*-values were listed in Table 1.

**Table 1:** The aberrantly expressed miRNAs in NAc from rats after treatment with alcohol. Source data is available for this figure in the Supporting Information

miRNAs	Vehicle	Alcohol	Alcohol/vehicle		miRNAs	Vehicle	Alcohol	Alcohol/vehicle	
							
Downregulated	Mean signal	Mean signal	% Change	*p*-value	Upregulated	Mean signal	Mean signal	% Change	*p*-value
rno-miR-379	3208	2309	72	2.24E-07	rno-miR-29b	274	4365	1595	6.37E-06
rno-miR-409-3p	684	487	71	8.23E-05	rno-miR-204	86	844	977	4.81E-05
rno-miR-181b	2734	1882	69	8.77E-04	rno-miR-27a	460	1682	366	2.17E-05
rno-miR-539	1991	1297	65	6.98E-10	rno-miR-335	562	2016	359	1.23E-04
rno-miR-25	896	583	65	3.51E-05	rno-miR-30a	1425	4401	309	8.15E-06
rno-miR-376b-5p	435	272	62	1.35E-04	rno-miR-29c	3350	9424	281	2.56E-05
rno-miR-15b	419	254	61	3.03E-05	rno-miR-101a	174	467	268	2.14E-05
rno-miR-382	4463	2621	59	7.13E-11	rno-miR-22	562	1467	261	1.04E-08
rno-miR-433	4454	2614	59	6.61E-11	rno-miR-30e	665	1662	250	8.09E-08
rno-miR-320	3629	2126	59	2.91E-12	rno-miR-100	1147	2089	182	1.43E-04
rno-miR-93	530	307	58	1.80E-08	rno-miR-99a	1374	2362	172	2.93E-04
rno-miR-151	5210	2994	57	1.16E-11	rno-miR-153	1316	2164	164	3.76E-06
rno-miR-361	7181	4126	57	1.27E-12	rno-miR-376b-3p	672	1072	160	3.79E-03
rno-miR-92b	4387	2436	56	5.07E-03	rno-miR-92a	644	985	153	5.42E-04
rno-miR-674-5p	1213	656	54	9.59E-11	rno-miR-145	1116	1666	149	1.90E-04
rno-miR-674-3p	455	245	54	3.74E-04	rno-miR-137	6651	9225	139	9.67E-06
rno-miR-129	671	357	53	5.66E-08	rno-miR-30b-5p	6317	8670	137	2.01E-04
rno-miR-338*	659	324	49	1.52E-08	rno-miR-212	1065	1434	135	3.65E-03
rno-miR-760-3p	501	238	47	8.29E-10	rno-miR-29a	16,672	22,118	133	1.41E-07
rno-miR-324-5p	836	376	45	3.20E-08	rno-miR-352	2631	3430	130	3.29E-03
rno-miR-330*	834	366	44	1.55E-13					
rno-miR-346	1086	448	41	1.81E-12					
rno-miR-485	1326	546	41	1.45E-13					
rno-let-7d*	1337	513	38	8.63E-08					
rno-miR-134	1214	362	30	9.10E-14					
rno-miR-122	687	87	13	5.66E-08					

Eighteen male rats (weighing 150–180 g) were divided into two treatment groups (*n* = 9): vehicle (500 µl saline, i.p. bid) or alcohol (1 g/kg, i.p. bid). Seven days later, the animals were sacrificed and their NAc were isolated for miRNA microarray analysis. miRNAs that were highly expressed in NAc and over 30% changes (*p* < 0.05, Student's *t*-test) in their expression after alcohol treatment were listed.

### Alcohol down-regulates the expression of miR-382 in rat NAc

**Figure 1:**
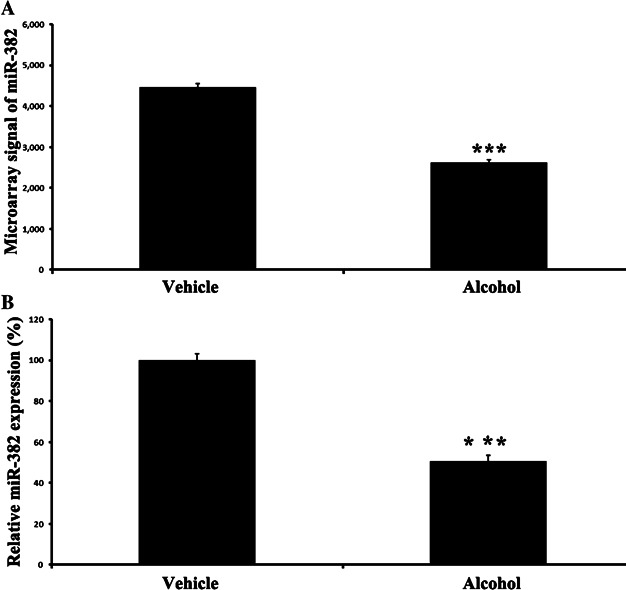
The expression of miR-382 in the nucleus accumbens (NAc) of rats after treatment with alcohol: 18 male rats were treated with vehicle (500 µl saline, i.p. bid) or alcohol (1 g/kg, i.p. bid) Seven days later, the animals were sacrificed and their NAc were isolated for miRNA microarray analysis, and miR-382 measurement by qRT-PCR. ***Student's *t*-test *p* < 0.0001, compared with that in vehicle-treated controls. Values are mean ± SEM from nine rats. miR-382 was decreased in alcohol-treated rats as determined by microarray analysis. *n* = 9, *p* = 1.93E-10.miR-382 was decreased in alcohol-treated rats as determined by qRT-PCR. *n* = 9, *p* = 6.53E-9.

Our microarray data revealed that miR-382 is an abundant miRNA in NAc and its expression was down-regulated after 7-days alcohol injection (*n* = 9, *t*-test *p* = 1.93E-10, compared with that in vehicle-treated controls) (Fig 1). To verify this important discovery, we further determined the levels of miR-382 with the qRT-PCR technique. Notably, miR-382 was decreased by about 50% in alcohol-treated rats, compared with that in vehicle-treated animals (*n* = 9, *t*-test, *p* = 6.53E-9) (Fig 1).

### miR-382 is a critical regulator for the signal molecules, DRD1 and DeltaFosB, in rat NAc

**Figure 2:**
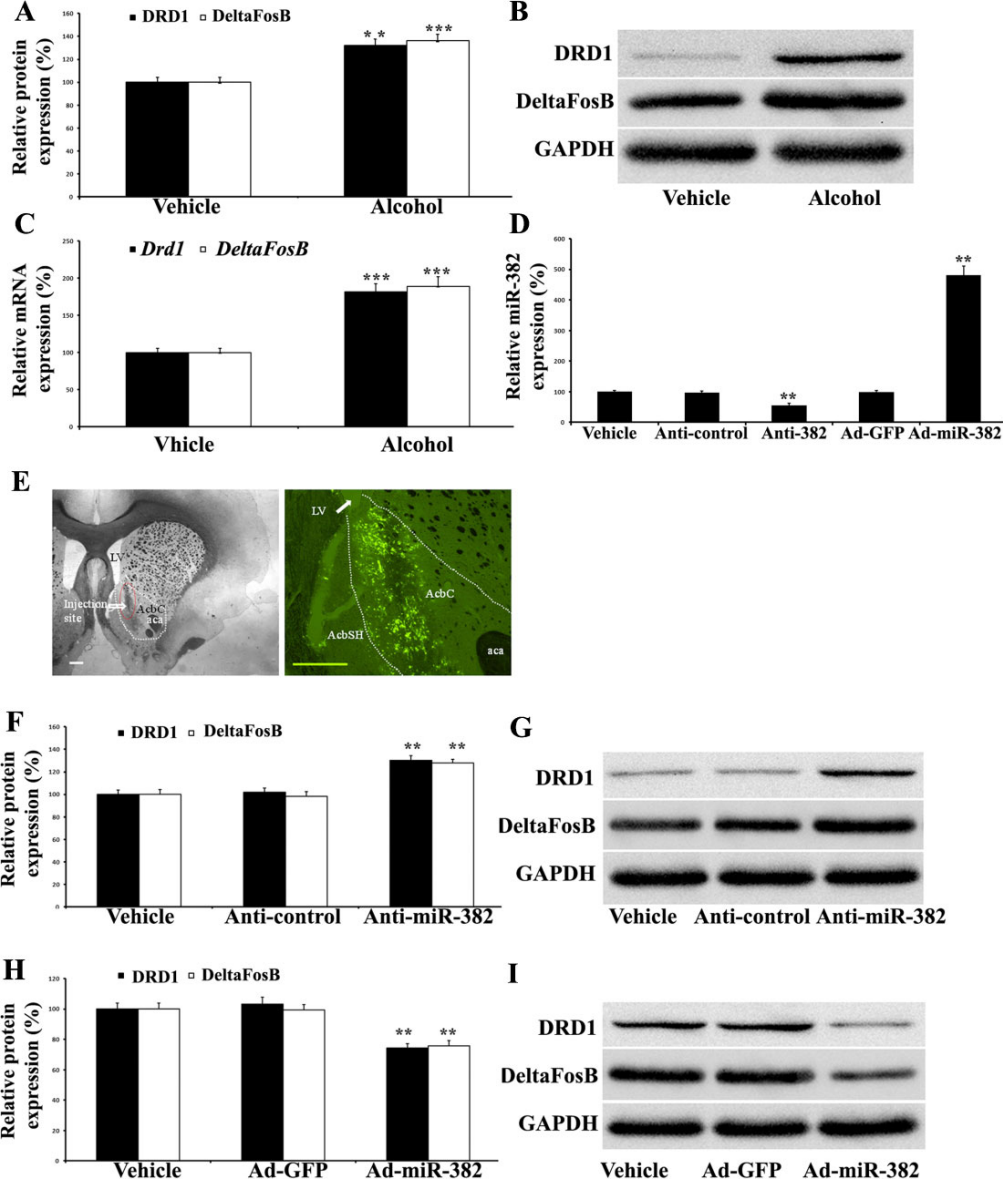
The effect of miR-382 on the expression of DRD1 and DeltaFosB in rat NAc: **p < 0.01, ***p < 0.001, Student's *t*-test Source data is available for this figure in the Supporting Information. Seven-days' alcohol injection (1 g/kg, i.p. bid) significantly increased the expression of DRD1 (*p* = 0.00106) and DeltaFosB (*p* = 0.00056) in rat NAc at the protein level. Values are mean ± SEM from 6 independent experiments (*n* = 6), compared with that in vehicle-treated controls.Representative Western blots of DRD1 and DeltaFosB from animals treated with vehicle or alcohol.Alcohol injections significantly increased the expression of DRD1 (*p* = 0.00022) and DeltaFosB (*p* = 0.00047) in rat NAc at the mRNA level. Values are mean ± SEM from 6 independent experiments (*n* = 6), compared with that in vehicle-treated controls.miR-382 expression in rat NAc was down-regulated by LNA-anti-miR-382 (Anti-miR-382) (*p* = 0.00133), but was up-regulated by Ad-miR-382 (*p* = 0.00102). Values are mean ± SEM from 4 independent experiments (*n* = 4), compared with that in the controls [LNA-anti-miR-382 control (Anti-control), and Ad-GFP].Left panel: view of injection site. Right panel: fluorescent image of GFP (green colour) in NAc at 5 days after microinjection of Ad-GFP. Injected Ad-GFP was limited to core region. Note: 40 µm slices, scale bar = 500 µm. aca, anterior commissure; AcBC, nucleus accumbens core; AabSH, nucleus accumbens shell; LV, lateral ventricle.The expression of DRD1 (*p* = 0.00057) and DeltaFosB (*p* = 0.0004) in rat NAc was increased by LNA-anti-miR-382. Values are mean ± SEM from 6 independent experiments (*n* = 6), compared with that in controls (Anti-control).Representative Western blots of DRD1 and DeltaFosB from animals treated with vehicle, anti-control or Anti-miR-382.Overexpression of miR-382 via Ad-miR-382 decreased the expression of DRD1 (*p* = 0.00041) and DeltaFosB (*p* = 0.00087) in rat NAc. Values are mean ± SEM from 6 independent experiments (*n* = 6), compared with that in control (Ad-GFP).Representative Western blots of DRD1 and DeltaFosB from animals treated with vehicle, Ad-GFP or Ad-miR-382.

To determine the potential role of miR-382 in alcohol intake, we determined the effect of miR-382 on the expression of DRD1 and DeltaFosB in rat NAc. As expected, 7-days' alcohol injection significantly increased the expression of DRD1 and DeltaFosB in rat NAc both at the protein (Fig 2) (DRD1, *n* = 6, *t*-test, *p* = 0.00106; DeltaFosB, *n* = 6, *t*-test, *p* = 0.00056) and mRNA levels (Fig 2) (*Drd1*, *n* = 6, *t*-test, *p* = 0.00022; *DeltaFosB*, *n* = 6, *t*-test, *p* = 0.00056). Representative Western blots of DRD1 and DeltaFosB from animals treated with vehicle or alcohol were shown in Fig 2.

To test the effects of miR-382 on the expression of DRD1 and DeltaFosB, both gain-of-function and loss-of-function approaches were applied. In this experiment, LNA-anti-miR-382 was used to knock-down its expression and Ad-miR-382 was used to up-regulate its expression. Vehicle control, LNA-anti-miR-382 control, adenovirus control (Ad-GFP), LNA-anti-miR-382 or Ad-miR-382, was injected into the NAc via microinjection. As shown in Fig 2, miR-382 level in rat NAc was successfully down-regulated by LNA-anti-miR-382 (*n* = 4, *t*-test, *p* = 0.00133), but was up-regulated by Ad-miR-382 (*n* = 4, *t*-test, *p* = 0.00102). To further confirm that the miRNA modulators we injected were located in the area of NAc, fluorescent signals of the injected Ad-GFP (green colour) were detected in brain sections by a fluorescent microscopy. As shown in Fig 2, the injected-adenoviruses were indeed localized in the area of NAc. In addition, the injected-Ad-miR-382 increased miR-382 expression in NAc, but not in the neighbouring brain areas (Supporting Information Fig S1).

Interestingly, the expression of DRD1 (*n* = 6, *t*-test, *p* = 0.00057) and DeltaFosB (Fig 2) (*n* = 6, *t*-test, *p* = 0.0004) in rat NAc was significantly increased by the LNA-anti-miR-382. Representative Western blots of DRD1 and DeltaFosB from animals treated with vehicle, anti-control or Anti-miR-382 were shown in Fig 2. In contrast, overexpression of miR-382 via Ad-miR-382 decreased the expression of DRD1 (*n* = 6, *t*-test, *p* = 0.00041) and DeltaFosB (*n* = 6, *t*-test, *p* = 0.00087) (Fig 2). Representative Western blots of DRD1 and DeltaFosB from animals treated with vehicle, Ad-GFP or Ad-miR-382 were shown in Fig 2. The results suggested that miR-382 is a critical regulator for both DRD1 and DeltaFosB in rat NAc.

### *Drd1* is a direct target gene of miR-382 and is a regulator for the expression of DeltaFosB

**Figure 3:**
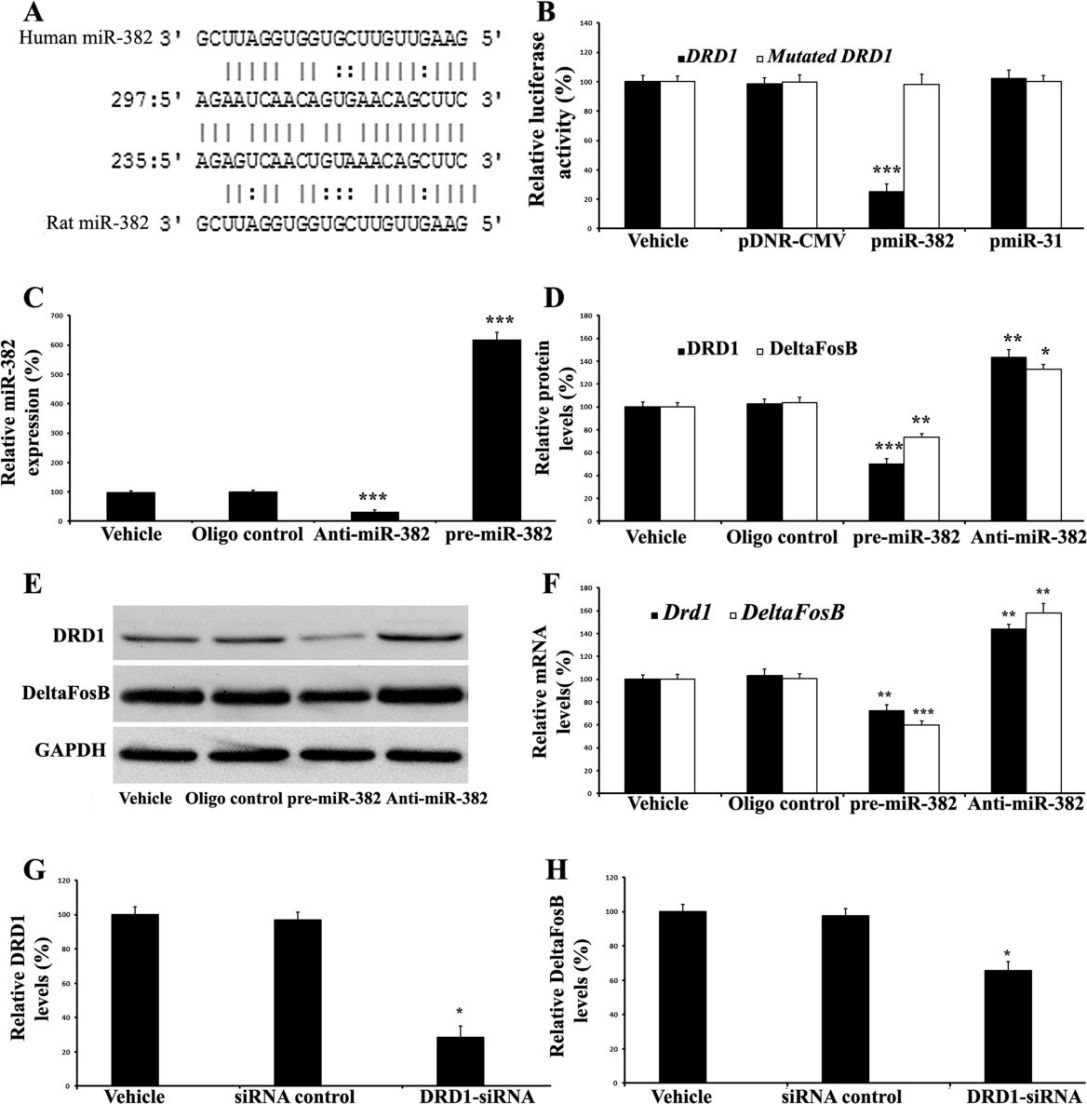
*Drd1* is a direct target gene of miR-382 and is a regulator for the expression of DeltaFosB in cultured CAD cells: **p < 0.01, ***p < 0.001, Student's *t*-test Source data is available for this figure in the Supporting Information. *Drd1* is a potential target gene of miR-382 predicted by computational analysis.The luciferase reporter construct, containing the putative miR-382 binding sequence from 3′-UTR of rat *Drd1* gene, was transfected into HEK293 cells with vehicle (Vehicle), an empty vector (pDNR-CMV), miR-382 (pmiR-31) or a control plasmid expressing an unrelated miRNA, miR-31 (pmiR-31). The construct with mutated fragment of the 3′-UTR of *Drd1* mRNA without the putative miR-382 binding sequences was used as the mutated control (mutated *Drd1*), pmiR-382, but not pmiR-31 or pDNR-CMV, inhibited luciferase activity (*p* = 1.17571599413E-6). Values are mean ± SEM from 6 independent experiments (*n* = 6), compared with that in control (pDNR-CMV). In the mutated control group, the inhibitory effect of pmiR-miR-382 on luciferase activity disappeared. Values are mean ± SEM from 3 independent experiments (*n* = 3), compared with that in control (pDNR-CMV).The expression of miR-382 in cultured CAD cells was modulated by LNA-anti-miR-382 (Anti-miR-382) and pre-miR-382. Vehicle and control oligos (oligo control) were used as controls. miR-382 expression was down-regulated by LNA-anti-miR-382 (*p* = 5.08726615734E-5), but was up-regulated by pre-miR-382 (*p* = 1.69645597989E-5). Values are mean ± SEM from 5 independent experiments (*n* = 5), compared with that in control (oligo control).At protein level, pre-miR-382 decreased the expression of DRD1 (*p* = 2.44969469509E-5) and DeltaFosB (*p* = 0.0008). In contrast, the expression of DRD1 (*p* = 0.00089) and DeltaFosB (*p* = 0.00149) was increased by LNA-anti-miR-382 (Anti-miR-382). Values are mean ± SEM from 5 independent experiments (*n* = 5), compared with that in oligo control.Representative Western blots of DRD1 and DeltaFosB.pre-miR-382 decreased, whereas Anti-miR-382 increased the expression of *Drd1* (*p* = 0.00416 and 0.00039) and *DeltaFosB* (*p* = 7.21292762637E-5 and 0.00027) at mRNA level. Values are mean ± SEM from 5 independent experiments (*n* = 5), compared with that in oligo control.DRD1 protein was knocked-down by its siRNAs (DRD1-siRNA) (*p* = 0.00137). Values are mean ± SEM from 3 independent experiments (*n* = 3), compared with that in siRNA control.DeltaFosB protein was decreased via knocking-down of DRD1 by its siRNAs (*p* = 0.00817). Values are mean ± SEM from 3 independent experiments (*n* = 3), compared with that in siRNA control.

Although miR-382 has a strong regulatory effect on the expression of DeltaFosB, computational analysis failed to find any binding sites for miR-382 in its mRNA sequence. Thus, *DeltaFosB* might not be a direct target gene of miR-382. We next performed the computational analysis on *Drd1* and found it has a miR-382 binding site at its 3′-UTR (Fig 3). The result suggests *Drd1* might be a potential direct target gene of miR-382.

Luciferase assay was performed to test whether or not the miR-382 could bind to and inhibit *Drd1*expressioin. As shown in Fig 3, miR-382 indeed could inhibit the luciferase activity (*n* = 6, *t*-test, *p* = 1.17571599413E-6). In the mutated control group, the inhibitory effect of miR-382 on luciferase activity in HEK 293 cells disappeared (Fig 3).

To further confirm that *Drd1* is a direct target gene in neuronal cells, the expression of miR-382 in cultured CAD cells was modulated by LNA-anti-miR-382 (*n* = 5, *t*-test, *p* = 5.08726615734E-5) and pre-miR-382 (*n* = 5, *t*-test, *p* = 1.69645597989E-5) (Fig 3). At the protein level, we found that pre-miR-382 decreased the expression of DRD1 (*n* = 5, *t*-test, *p* = 2.44969469509E-5) and DeltaFosB (*n* = 5, *t*-test, *p* = 0.0008). In contrast, the expression of DRD1 (*n* = 5, *t*-test, *p* = 0.00089) and DeltaFosB (*n* = 5, *t*-test, *p* = 0.00149) was increased by LNA-anti-miR-382 (Anti-miR-382) (Fig 3). Representative Western blots of DRD1 and DeltaFosB were shown in Fig 3). At the mRNA level, we also found that pre-miR-382 decreased, whereas Anti-miR-382 increased the expression of *Drd1* (*n* = 5, *t*-test, *p* = 0.00416 and *n* = 5, *t*-test, *p* = 0.00039) and *DeltaFosB* (*n* = 5, *t*-test, *p* = 7.21292762637E-5 and *n* = 5, *t*-test, *p* = 0.00027) (Fig 3). The results suggest that DeltaFosB might be a downstream signal molecule of DRD1. To further verify this discovery, the expression DRD1 in CAD cells was knocked-down by its siRNAs (*n* = 3, *t*-test, *p* = 0.00137) (Fig 3). As expected, the expression of DeltaFosB was down-regulated by inhibition of DRD1 (*n* = 3, *t*-test, *p* = 0.00817) (Fig 3).

### Overexpression of miR-382 via adenovirus-mediated gene transfer is sufficient to inhibit alcohol-induced up-regulation of the DRD1 and DeltaFosB in rat NAc

This experiment is for answering an important question: whether or not the alcohol-induced up-regulation of DRD1 and DeltaFosB can be prevented by overexpression of miR-382. Three days before alcohol administration, 4 µl of vehicle, Ad-GFP or Ad-miR-382 (1 × 10^9^ pfu/ml) was infused into the NAc of rats. Then, the animals were divided into the following groups: vehicle-treated rats without alcohol administration (vehicle group); Ad-GFP-treated rats without alcohol administration (Ad-GFP group); Ad-GFP-treated rats with alcohol administration (Alcohol + Ad-GFP group) and Ad-miR-382-treated rats with alcohol administration (Alcohol + Ad-miR-382 group). Severn days later, the rat NAc were isolated.

**Figure 4:**
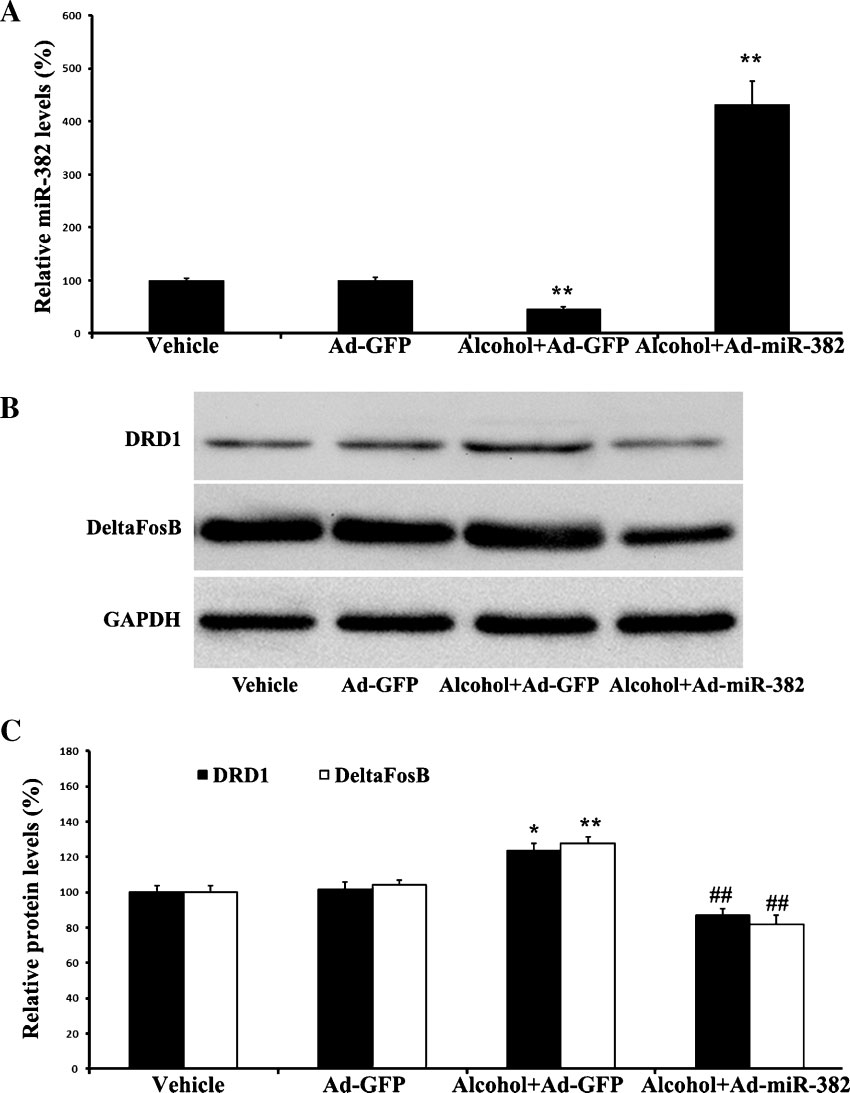
Overexpression of miR-382 is sufficient to inhibit alcohol-induced up-regulation of DRD1 and DeltaFosB in rat NAc: *p < 0.01, **p < 0.001, ##*p* < 0.001, Student's *t*-test Source data is available for this figure in the Supporting Information. At 3 days before alcohol administration, 4 µl of vehicle, Ad-GFP or Ad-miR-382 (1 × 10^9^ pfu/ml) was infused into the NAc of rats. Then, the animals were divided into the following groups: Vehicle-treated rats without alcohol administration (Vehicle); Ad-GFP-treated rats without alcohol administration (Ad-GFP); Ad-GFP-treated rats with alcohol administration (Alcohol + Ad-GFP) and Ad-miR-382-treated rats with alcohol administration (Alcohol + Ad-miR-382). Severn days later, the rat NAc were isolated. The successful modulation of miR-382 expression by Ad-miR-382. Alcohol decreased the expression of miR-382 in rat NAc (*p* = 0.00076). Values are mean ± SEM from 3 independent experiments (*n* = 3), Alcohol + Ad-GFP compared with that in Ad-GFP control. Ad-miR-382 increased the expression of miR-382 in NAc of rats with alcohol administration (*p* = 0.00079). Values are mean ± SEM from 3 independent experiments (*n* = 3), Alcohol + Ad-miR-382 compared with that in Alcohol + Ad-GFP control.Representative Western blots in rat NAc from different treatments.Downregulation of DRD1 and DeltaFosB via overexpression of miR-382 in NAc. Alcohol administration increased the expression of DRD1 (**p = 0.00616) and DeltaFosB (**p = 0.00087). Values are mean ± SEM from 5 independent experiments (*n* = 5), Alcohol + Ad-GFP compared with that in Ad-GFP control. Overexpression of miR-382 via Ad-miR-382 prevented alcohol-induced up-regulation of DRD1 (##*p* = 0.0002) and DeltaFosB (##*p* = 0.00012) in rat NAc. Values are mean ± SEM from 5 independent experiments (*n* = 5), Alcohol + Ad-miR-382 compared with that in Alcohol + Ad-GFP control.

The successful up-regulation of miR-382 via Ad-miR-382 was verified by qRT-PCR (*n* = 3, *t*-test, *p* = 0.00076) (Fig 4). Representative Western blots in rat NAc from different treatments were shown in Fig 4. Clearly, alcohol administration increased the expression of DRD1 (*n* = 5, *t*-test, *p* = 0.00616) and DeltaFosB (*n* = 5, *t*-test, *p* = 0.00087) (note: Alcohol + Ad-GFP group compared with Ad-GFP group). However, the alcohol-induced up-regulation of DRD1 (*n* = 5, *t*-test, *p* = 0.0002) and DeltaFosB (*n* = 5, *t*-test, *p* = 0.00012) was prevented by the overexpression of miR-382 (note: Alcohol + Ad-miR-382 group compared with Alcohol + Ad-GFP group) (Fig 4).

### Overexpression of miR-382 via Ad-miR-382 is sufficient to inhibit the voluntary intake of and the preference for alcohol in rats under the intermittent access two-bottle choice drinking paradigm

**Figure 5:**
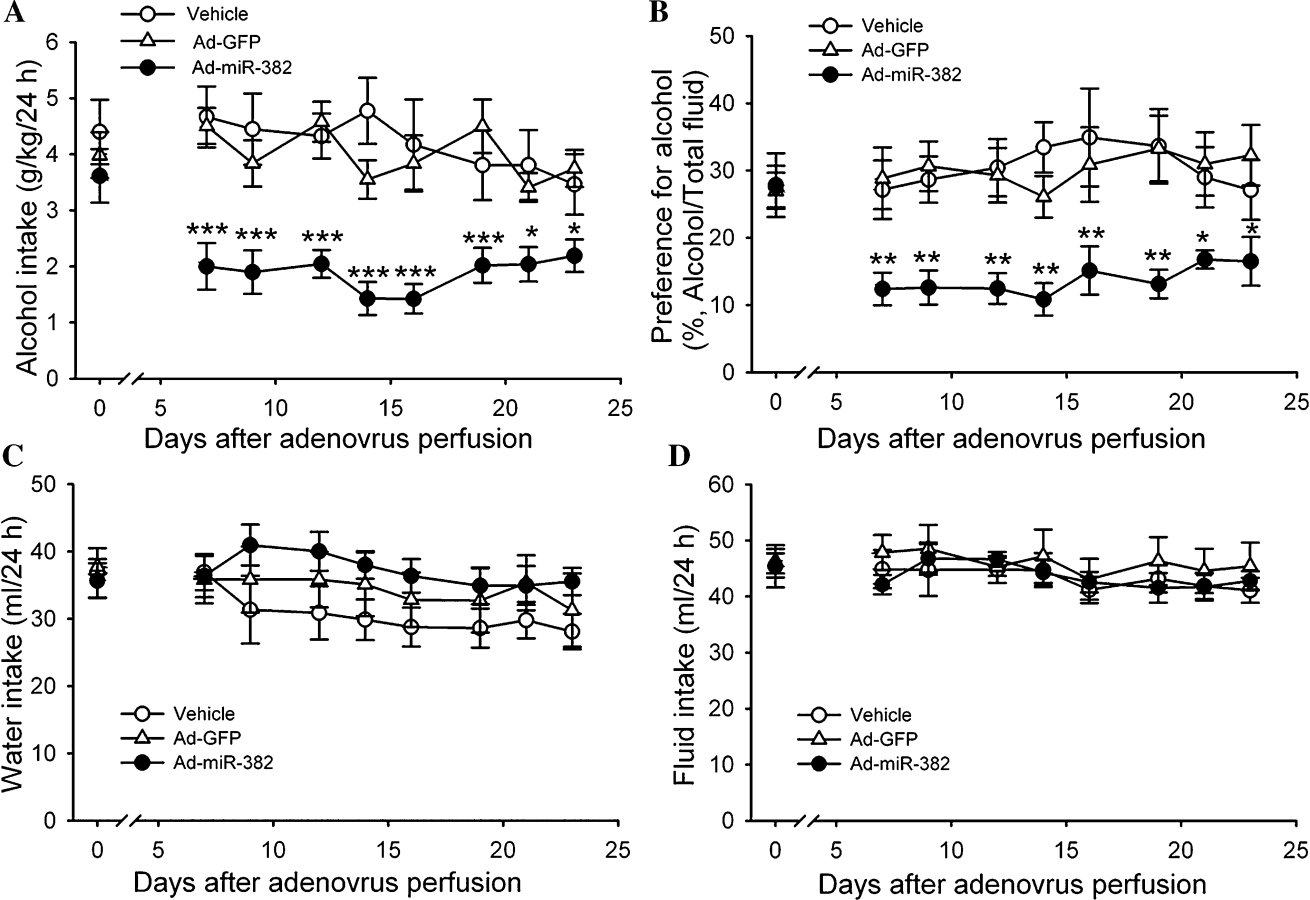
Overexpression of miR-382 is sufficient to inhibit the voluntary intake of and the preference for alcohol in rats: the animals (total *n* = 24) under the intermittent access two-bottle choice drinking paradigm were randomly divided into three groups which received infusion of Ad-miR-382, control adenovirus Ad-GFP or vehicle (saline), respectively, into the NAc Seven days later, the rats were allowed to resume ethanol consumption under the same drinking paradigm. The successful up-regulation of miR-382 via Ad-miR-382 was verified by qRT-PCR at 7 days after drinking (Supporting Information Fig S1). The successful modulation of DRD1 and DeltaFosB via Ad-miR-382 was verified by Western blot analysis at 7 days after drinking (Supporting Information Fig S2). The voluntary intake of alcohol was reduced via Ad-miR-382 at all points (7 days: *p* = 0.00039; 9 days: *p* = 0.00046; 11 days: *p* = 0.00057; 13 days: *p* = 0.00026; 17 days: *p* = 0.00018; 19 days: *p* = 0.00098; 21 days: *p* = 0.013; 23 days: *p* = 0.0035).Ad-miR-382 decreased the preference for alcohol at all points (7 days: *p* = 0.0037; 9 days: *p* = 0.0041; 11 days: *p* = 0.0029; 13 days: *p* = 0.0015; 17 days: *p* = 0.0088; 19 days: *p* = 0.0068; 21 days: *p* = 0.018; 23 days: *p* = 0.0029).Ad-miR-382 did not alter the water consumption.Ad-miR-382 did not alter the total fluid intake. Values are mean ± SEM from 8 independent experiments (*n* = 8), compared with that in Ad-GFP control. *p < 0.05, **p < 0.01 ***and *p* < 0.001, two-way ANOVA with repeated measure.

This experiment was to determine whether the changes of miR-382 expression in the NAc could affect the drinking behaviour. Male rats (*n* = 24) were first trained to drink 20% ethanol under the intermittent access two-bottle choice paradigm. When rats maintained a stable baseline level of alcohol (ethanol) consumption (4.3 ± 0.2 g/kg/24 h) for 4 weeks (12 drinking sessions in total), they were randomly divided into three groups which received infusion of Ad-miR-382, control adenovirus Ad-GFP or vehicle (saline), respectively, into the NAc as described above. The successful up-regulation of miR-382 via Ad-miR-382 was verified by qRT-PCR at 7 days after alcohol drinking (Supporting Information Fig S2). The successful modulation of DRD1 and DeltaFosB via Ad-miR-382 was verified by Western blot analysis at 7 days after alcohol drinking (Supporting Information Fig S3). Seven days later, the rats were allowed to resume alcohol (ethanol) consumption under the same drinking paradigm. As illustrated in Fig 5, in the group which received Ad-miR-382, the voluntary intake of alcohol was significantly reduced, as demonstrated by the decreased overall main effect of treatment, main effect of day, and the significant treatment by day interaction on alcohol consumption. The reduction of alcohol intake started on the 7th day after Ad-miR-382 infusion into the NAc, and sustained during the 18-day period of observation (*n* = 8, two-way ANOVA, 7 days: *p* = 0.00039; 9 days: *p* = 0.00046; 11 days: *p* = 0.00057; 13 days: *p* = 0.00026; 17 days: *p* = 0.00018; 19 days: *p* = 0.00098; 21 days: *p* = 0.013; 23 days: *p* = 0.0035) (Fig 5). In addition, the reduction of alcohol intake was paralleled by a significant decrease in the preference for alcohol during the 18-day period of observation (*n* = 8, two-way ANOVA, 7 days: *p* = 0.0037; 9 days: *p* = 0.0041; 11 days: *p* = 0.0029; 13 days: *p* = 0.0015; 17 days: *p* = 0.0088; 19 days: *p* = 0.0068; 21 days: *p* = 0.018; 23 days: *p* = 0.0029) (Fig 5). In contrast, the control adenovirus, Ad-GFP had no effect on either the intake of (Fig 5) or the preference for alcohol (ethanol) (Fig 5). Interestingly, Ad-miR-382 did not significantly alter the water consumption (Fig 5) or the total fluid intake (Fig 5).

### Overexpression of miR-382 influences responses of MSNs in NAc slices to DRD1 activation

**Figure 6:**
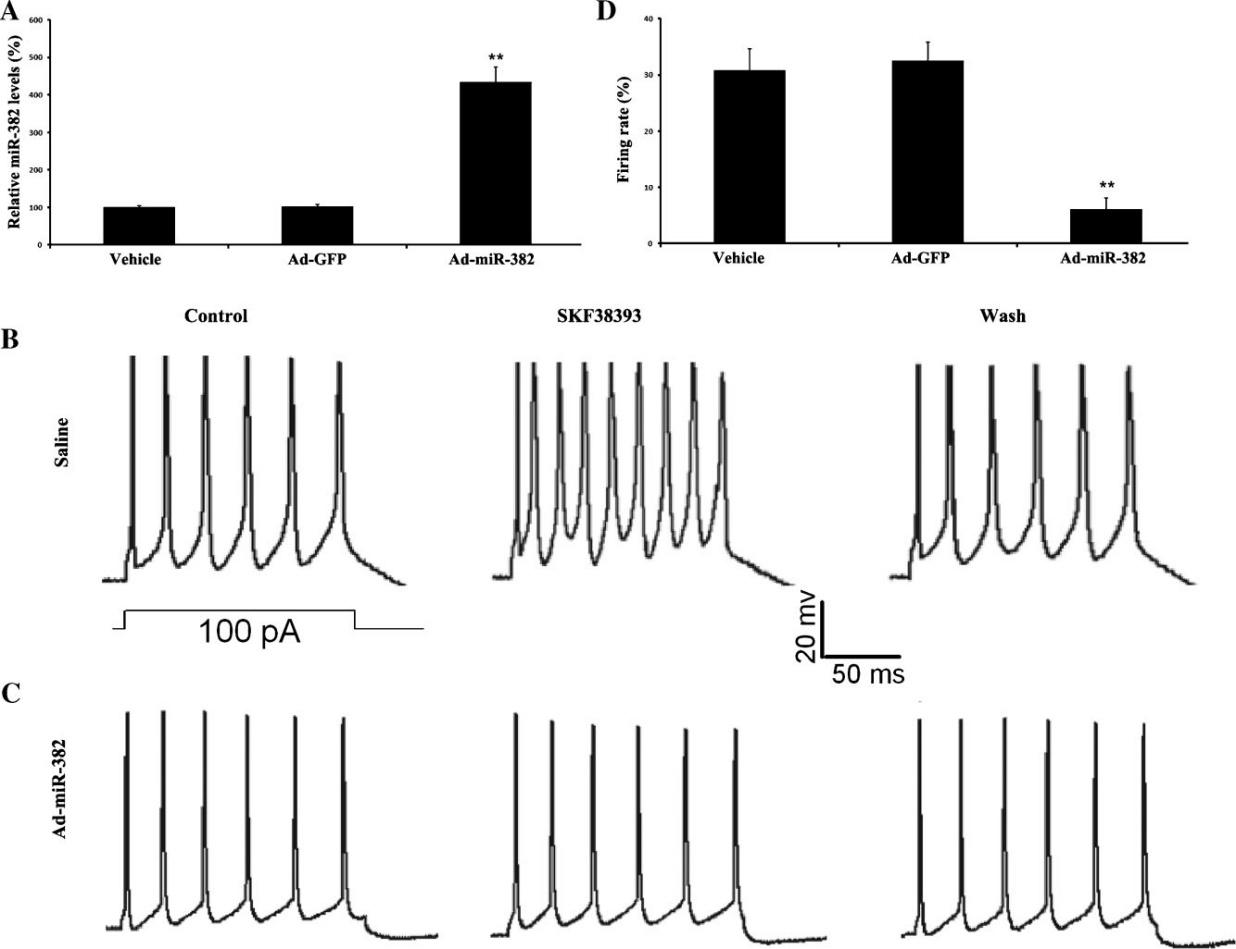
Overexpression of miR-382 influences responses of MSNs in NAc slices to DRD1 activation: 4 µl of vehicle, Ad-GFP or Ad-miR-382 (1 × 10^9^ pfu/ml) was infused into the NAc of rats Severn days later, the NAc were isolated. **A-C.** The successful modulation of miR-382 expression by Ad-miR-382. Ad-miR-382 increased the expression of miR-382 in NAc of rats (*p* = 0.00125). Values are mean ± SEM from 3 independent experiments (*n* = 3), compared with that in Ad-GFP control. *p < 0.01, Student's *t*-test. Sample voltage traces in response to current injections (inset) from a MSN in acute brain slices of rats which received infusion of saline (B) and Ad-miR-382 (C), respectively. While the application of 1 µM SKF38393, the DRD1 agonist increased the firing rate of the MSN of rats that received saline injection, this was not seen in rats received Ad-miR-382 injection.**D.** Summary graphs showing that Ad-miR-382 NAc injection attenuated the firing rate of MSNs–induced by 1 µM SKF38393 (*p* = 0.00654). Values are mean ± SEM from 4 independent experiments (*n* = 4), compared with that in Ad-GFP control. **p < 0.01, two-way ANOVA.

The above experiments indicate that miR-382 in the NAc has a particularly critical role in mediating the behavioural responses to alcohol. To determine the potential cellular modifications via miR-382 that could cause its behavioural effects, male rats (*n* = 12) were randomly divided into three groups (*n* = 4 for each) which received infusion of Ad-miR-382, control adenovirus Ad-GFP or vehicle, respectively, into the NAc as described above. Seven days later, the rats were sacrificed, their brains were removed and brain slices were prepared. The successful up-regulation of miR-382 via Ad-miR-382 was verified by qRT-PCR (*n* = 3, *t*-test, *p* = 0.00125) (Fig 6). Electrical activities were recorded using whole-cell recordings in current-clamp mode in MSNs from the NAc in acute brain slices. When compared to cells in saline injected rats, neither Ad-miR-382, nor control adenovirus Ad-GFP altered the MSNs' passive membrane properties, resting potential or action potential waveform (Supporting Information Table S2). We next tested their response to DRD1 activation. The application of the DRD1 agonist of SKF38393 (1 µM) increased the action potential responses to current injection of the MSNs in rats that received NAc injections of either the control adenovirus Ad-GFP or the vehicle, but not in rats that received Ad-miR-382 injection (*n* = 4, two-way ANOVA, *p* = 0.00654) (Fig 6).

## DISCUSSION

It is well established that although the original addictive drugs, reagents or factors are diverse, in view of molecular mechanisms, they could finally induce addiction by modulating the genes in the brain (Nestler, 2005). Indeed, alcohol addiction is a complex process in which many genes such as Drd1 and *DeltaFosB* are involved (Camarini et al, 2011; Marttila et al, 2007; Morikawa and Morrisett, 2010). However, how these abuse-related genes are regulated under the condition of alcohol intake is still unclear.

MiRNAs are a new layer of gene regulators that directly regulate over 30% of genes in a cell via degradation or translational inhibition of their target mRNAs (Ambros, 2004; Lewis et al, 2005). Moreover, much more genes can also be regulated indirectly by miRNAs. Thus, it is reasonable that miRNAs may play important roles in the regulation of abuse-related genes and in the pathogenesis of alcohol addiction. The potential involvement of miRNAs in alcohol intake was suggested by the following two recent reports: In cultured rodent neurons, alcohol is found to be able to up-regulate the miR-9 and down-regulate the expression of its target gene, alpha subunit of BK channel, which is a molecule related to the alcohol addiction (Pietrzykowski et al, 2008). Another report demonstrated that the artificial microRNA-based neurokinin-1 receptor gene silencing reduces alcohol consumption in mice (Baek et al, 2010).

To provide the direct evidence that miRNAs are involved in alcohol intake, we determined the expression profile of miRNAs in rat NAc after treatment with alcohol. The results clearly showed that multiple miRNAs were aberrantly expressed in NAc from alcohol-treated rats. Among them, miR-382 was found to be down regulated by about 50% after treatment with alcohol.

To determine the potential role of miR-382 in alcohol intake, the effect of miR-382 on the expression of DRD1 and DeltaFosB in rat NAc was determined. The expression of both DRD1 and DeltaFosB in rat NAc was significantly up-regulated after treatment with alcohol. Both gain-of-function and loss-of-function approaches have demonstrated that miR-382 was a strong regulator for the expression of both DRD1 and DeltaFosB in rat NAc. Moreover, the alcohol-mediated up-regulation of DRD1 and DeltaFosB was significantly inhibited by overexpression of miR-382.

DRD1 and DeltaFosB are two critical molecules related to alcohol abuse as described in Section 1. Our computational analysis and experimental approach have demonstrated that *Drd1* might be a direct target gene of miR-382. However, the computational analysis failed to find any binding sites of miR-382 in the mRNA of *DeltaFosB*. The result indicates that *DeltaFosB* might not be a direct target gene for miR-382. Based on the results, we hypothesize that DRD1 might be an upstream signal molecule for DeltaFosB and miR-382-mediated effect on the expression of DeltaFosB might be induced by DRD1. This hypothesis is supported by the following studies: first, DRD1 activation via abuse-related drugs or DRD1 agonists is able to increase the expression of DeltaFosB (Chocyk et al, 2006; Doucet et al, 1996; Hu et al, 2002; Kim et al, 2009). Second, the addictive psychostimulant-induced expression of DeltaFosB can be attenuated or abolished by DRD1 antagonist (Chocyk et al, 2006; Muller and Unterwald, 2005; Sun et al, 2008). In addition, dopamine precursor molecule 3,4-dihydroxyphenyl-l-alanine (l-DOPA)-induced dyskinesias and up-regulation of DeltaFosB can be blocked by genetic inactivation of DRD1 (Darmopil et al, 2009). Moreover, DRD1-induced events such as priming can be effectively inhibited by knockdown of DeltaFosB (Crocker et al, 1998). To further verify that the DRD1 is an upstream molecule of DeltaFosB, the expression of DRD1 was knocked-down in cultured CAD cells via its siRNA. As expected, the expression of DeltaFosB was significantly inhibited in these cells (Fig 3).

The role of miR-382 in alcohol intake was determined in rats using an intermittent access two-bottle choice drinking paradigm. We found that overexpression of miR-382 in rat NAc via adenovirus-mediated gene transfer is sufficient to inhibit the voluntary intake of and preference for alcohol in these rats.

The NAc plays critical roles in many drugs of abuse including alcohol (Cadoni and Di Chiara, 2007; Goto and Grace, 2008; Nestler, 2001). It is well-known that dopamine is a fundamental modulator of NAc MSN activity via both the D1-like (D1R) and D2-like (D2R) DA receptors (Hara and Pickel, 2005). In the current study, we showed that SKF38393 increased the firing of NAc MSNs using the patch clamp technique, which is consistent with recent finding that DRD1 activation is able to induce membrane depolarization (Podda et al, 2010). Although DRD1 was still expressed at very low level in miR-382-overexprssed NAc, the changes in excitability of NAc MSNs caused by DRD1 activation had been completely blocked. We think that there are two possibilities for this discrepancy. First, the changes in excitability of NAc MSNs may require a significant amount of basal DRD1. Second, although miR-382-DRD1 is a critical pathway for DRD1-mediated effect on MSN firing, miR-382 may also have other unidentified targets that could also affect the DRD1 agonist-mediated effect on MSN excitability.

Advances in the fields of genomics and genetics in the last decade have identified a large number of genes that can potentially influence alcohol-drinking behaviour in humans as well as animal models. However, it is still unclear how these genes are regulated under the conditions of alcohol use and abuse. The discovery of miRNAs and their fine-tuned control of multiple gene expression at the post-transcription level may represent an important molecular mechanism responsible for alcohol abuse-related neuroadaptive changes at functional, neurochemical and structural levels (Miranda et al, 2010). However, the detailed effect of individual miRNA on alcohol intake/abuse and its target genes still need to be defined.

In summary, the expression profile of miRNAs in rat NAc after treatment with alcohol was identified. The results suggest that multiple miRNAs may be involved in alcohol-mediated gene expression in NAc. Among them, miR-382 is an important regulator and participator in alcohol intake via its direct target gene Drd1 and its downstream signal molecule DeltaFosB. MiRNAs may be novel therapeutic targets for alcohol addiction.

## MATERIALS AND METHODS

### Animals

Male Sprague-Dawley rats (weight 150–180 g at the start of the experiments) were used for this experiment. All animals were housed in separate cages in 12-h light and 12-h dark periods with free access to water and food. The animal protocol was approved by the Institutional Animal Care and Use Committee and was consistent with the Guide for the Care and Use of Laboratory Animals (NIH publication 85–23, revised 1985).

### Administration of alcohol

Rats were treated with vehicle (saline) or alcohol (ethanol) (1 g/kg) by intraperitoneal (i.p.) injection twice per day for seven days.

### Generation of the adenovirus expressing miR-382 (Ad-miR-382) and control adenovirus expressing GFP (Ad-GFP)

Ad-miR-382 and Ad-GFP were generated using ViraPower™ Adenoviral Gateway Expression system (Invitrogen, CA) according to the manufacturer's protocols as described previously (Cheng et al, 2009).

### Regulation of the miR-382 expression in rat NAc via microinjection

The expression of miR-382 in rat NAc was down-regulated via its specific antisense, the locked-nucleic-acid-modified antisense oligonucleotide for miR-382 (LNA-anti-miRNA-382), and was up-regulated via Ad-miR-382. LNA-anti-miR-382 and its negative random oligonucleotide control (LNA-anti-miR-382 control) were synthesized by Integrated DNA Technologies. The sequence of the LNA-anti-miR-382 is: 5′mCmGmAmAmUmCmCmAmCmCmAmCmGmAmAmCmAmAmCmUmUmC-3′. LNA-anti-miR-382, Ad-miR-382 or their controls (vehicle, LNA-anti-miR-382 control, or Ad-GFP) was injected into the NAc via microinjection. Briefly, the rats were anesthetized with ketamine (80 mg/kg, i.p.) and were placed in a stereotaxic apparatus with the incisor bar set at 3.3 mm below the interaural line. Two small holes were made in the skull using a dental drill. The vehicle (4 µl saline), LNA-anti-miR-382 (4 µl, 100 µM in 1% transfection reagent), LNA-LNA-anti-miR-382 control (4 µl, 100 µM in 1% transfection reagent), Ad-miR-382 (4 µl, 1 × 10^9^ pfu/ml) or the control virus (4µl, Ad-GFP) was injected bilaterally into NAc core with two 10 µl Hamilton syringe at a rate of 0.1 µl/min. The incisions were closed after treatments.

### Sacrifice of animals, tissue preparation and fluorescent images

The rats were sacrificed by decapitation at the end of experiments. Rat brains were rapidly isolated and the frozen brain sections included the core and shell subdivisions of the NAc were cut as described previously (Xiong et al, 2011). To detect the location of the injected adenovirus, the fluorescent signals of the adenovirus-expressed GFP were determined in brain sections by a fluorescent microscopy (Nikon) equipped with a CCD camera and image software.

### Catecholaminergic neuronal cell (CAD cell) culture, the expression modulation of miR-382 and knockdown of DRD1

CAD cells were grown in DMEM/F-12 medium, supplemented with 8% FBS and 1% penicillin–streptomycin on standard tissue culture dishes in a humidified 5% CO_2_ incubator. CAD cells were passaged every 3–4 days by pipetting cells from a confluent plate and triturating them in 5 ml of fresh medium.

Oligo transfection was performed according to the established protocol (Cheng et al, 2009, Liu et al, 2009; Ji et al, 2009). Briefly, cells were transfected using a transfection reagent (Qiagen) at 24 h after seeding into the well. Transfection complexes were prepared according to the manufacturer's instructions. For the miR-382 knockdown, LNA-anti-miR-382 was added to the culture media at final oligonucleotide concentration of 50 nM. For the miR-382 up-regulation, pre-miR-382 (Ambion, Inc.) was added directly to the complexes at final oligonucleotide concentration of 10 nM. Vehicle control, and oligo controls for LNA-anti-miR-382 (LNA-anti-miR-382 control) and pre-miR-382 (pre-miR-382 control) were used as controls. To knock-down the DRD1 in CAD cells, the siRNA of DRD1 (DRD1-siRNA, 50 nM) or its negative control (siRNA-control, 50 nM) (Santa Cruz Biotechnology) was added into cultured medium.

### RNA isolation and qRT-PCR

RNAs levels were isolated and determined by qRT-PCR as described previously (Liu et al, 2011; Liu et al, 2012). As an internal control, U6 was used for miR-382 template normalization and GAPDH was used for *DeltaFosB* and *Drd1* template normalization. The PCR primer sequences are listed in Supporting Information Table S1.

**The paper explained**

PROBLEM:

Alcohol addiction is a major social and health concern. Because of the limited understanding of the underlying causes of alcohol addiction, effective strategies for treating alcoholism are still lacking. There is an urgent need to improve our understanding of the molecular mechanisms of alcohol addiction and to develop novel therapeutic strategies for this complex disorder. MicroRNAs (miRNAs) are a novel class of noncoding RNAs that negatively regulate over 30% of genes in a cell with strong biological functions. However, the roles of miRNAs in alcohol addiction are still unclear. The aim of this study is to determine the expression profile of miRNAs in the nucleus accumbens (NAc) of rats treated with alcohol, and the role of an alcohol-regulated miR-382 in alcohol intake as well as the potential molecular mechanisms involved.

RESULTS:

The miRNA microarray analysis has revealed that multiple miRNAs are aberrantly expressed in the NAc of rats after treatment with alcohol. Among them, miR-382 is down-regulated by about 50% in alcohol-treated rats. To determine the biological functions of miR-382, its expression is up-regulated by adenovirus expressing miR-382 (Ad-miR-382) or pre-miR-382, but is down-regulated by its special antisense oligonucleotide (LNA-anti-miRNA-382). In both cultured neuronal cells *in vitro* and in the brain NAc *in vivo*, we have identified that the dopamine receptor D1 (DRD1), an alcohol addiction-related signal molecule, is a direct target gene of miR-382. Via this target gene, miR-382 strongly modulates the expression of another alcohol-related signal molecule, DeltaFosB. Moreover, overexpression of miR-382 significantly attenuates alcohol-induced upregulation of DRD1 and DeltaFosB, decreases voluntary intake of and preference for alcohol, and inhibits the DRD1-induced action potential responses.

IMPACT:

The expression profile of miRNAs in rat NAc after chronic treatment with alcohol is identified. The results suggest that multiple miRNAs may be involved in alcohol-mediated gene expression in NAc. Among them, miR-382 is an important regulator and participator in alcohol intake via its direct target gene *Drd1* and its downstream signal molecule DeltaFosB. miRNAs may be novel therapeutic targets for alcohol addiction.

### Western blot analysis

Proteins isolated from NAc and CAD cells were determined by Western blot analysis. Equal amounts of protein were subjected to SDS-PAGE. A standard Western blot analysis was conducted using DeltaFosB rabbit monoclonal antibody (1:100 dilution; Cell Signalling) and DRD1 rabbit polyclonal antibody (1:1000 dilution; Proteintech) GAPDH antibody (1:5000 dilution; Cell Signalling) was used as a loading control.

### miRNA microarray analysis

After treatment with alcohol and saline for 7 days, miRNAs were isolated from the NAc of rats using the mirVana miRNA isolation kit (Ambion, Inc.). MiRNA expression was determined by miRNA microarray analysis as described previously (Cheng et al, 2007; Ji et al, 2009). Each group had nine rats, and miRNA expression profiling was done by miRNA microarray analysis using a chip containing 300 mature miRNAs (Chip ID miRRat 9.2 version; LC Sciences). The microarray data from this publication have been submitted to the GEO (http://www.ncbi.nlm.nih.gov/geo/) and assigned the identifier GSE47943.

### Luciferase assay

The reporter plasmid, a firefly luciferase reporter construct psiCHECK-2 (Promega, WI) inserted with a fragment of the 3′-UTR of rat *Drd1* mRNA containing the putative miR-382 binding sequence. The construct with mutated fragment of the 3′-UTR of *Drd1* mRNA without the putative miR-382 binding sequences was used as the mutated control. HEK 293 cells were transfected with the construct or the mutated control construct. Then, these HEK 293 cells were treated with vehicle, pDNR-CMV (an empty plasmid, 0.2 µg/ml), pmiR-382 (a plasmid expressing miR-382, 0.2 µg/ml) or pmiR-31 (a plasmid expressing miR-31, 0.2 µg/ml). Forty-eight hours after treatment, cell extract was isolated to measure the luciferase expression on a scintillation counter by using a dual luciferase reporter system (Promega, WI).

### The alcohol intake and preference assay in rats

The alcohol intake and preference were determined by the two-bottle, intermittent alcohol access choice drinking model as described previously (Li et al, 2010; Simms et al, 2008). In Brief, on Monday, Wednesday and Friday, the rats were given 24-h concurrent access to two bottles, one with alcohol (20% v/v) and the other with water. The rats had unlimited access to two bottles of water on the remaining days of each week. Bottle position was randomly assigned for each alcohol drinking session. The amount of alcohol consumed was determined by bottle weight (g) before and after the 24 h of alcohol access, and the water consumed was determined by water volume before and after the 24 h of access.

After 18 alcohol access sessions, vehicle (4 µl saline), control adenovirus Ad-GFP (4 µl, 1 × 10^9^ pfu/ml) or Ad-miR-382 (4 µl, 1 × 10^9^ pfu/ml) was injected into rat NAc via microinjection as described. Seven days after treatments, rats were again allowed access to the intermittent-access 20% alcohol procedure for seven drinking sessions.

### Slice preparation for electrophysiology recordings

The brain slices were cut from the anesthetized and decapitated Sprague-Dawley rats using a VF-200 slicer as described previously (Jiang et al, 2004; Ye et al, 2006). These brain slices were prepared in the ice-cold glycerol-based artificial cerebrospinal fluid (ACSF) (carbogen) and were allowed to recover for at least 1 h before the electrophysiology recordings at 32°C in carbogen-saturated regular ACSF containing 125 mM NaCl, 1.6 mM KCl, 1.2 mM NaH_2_PO_4_, 1.2 mM MgCl_2_, 2.4 mM CaCl_2_, 25 mM NaHCO_3_ and 11 mM glucose, and saturated with 95%O_2_/5%CO_2_ (Ye et al, 2006).

### Patch-clamp electrophysiology

Whole-cell recordings from medium spiny neurons (MSNs) located in the NAc shell and core were obtained under visual control on a differential interference contrast, upright microscope with infrared illumination (Ye et al, 2006). Signals were recorded with MultiClamp 700A amplifiers (Axon Instruments, Forster City, CA), a Digidata 1320A A/D converter (Axon Instruments) and pCLAMP 9.2 software (Axon Instruments). Data were filtered at 1 kHz and sampled at 5 kHz. A single slice was transferred to the 0.4 ml recording chamber, where it was stabilized by a platinum ring. Throughout the experiments, the bath was continually perfused with carbogenated ACSF (1.5–2.0 mL/min). All recordings were made at 32°C, maintained by an automatic temperature controller. Cells were visualized with an upright microscope and near-infrared illumination.

### Statistical analysis

All data are expressed as mean ± SEM (standard error of the mean). All the experiments were repeated independently at least three times except for the behavioural experiments. For relative gene expression, the mean value of vehicle control group is defined as 100%. Two-tailed unpaired Student's *t*-tests and ANOVA were used for statistical evaluation of the data. Behavioural data were analysed using two-way ANOVA with repeated measure, followed by Student-Newman-Keuls *post hoc* test when indicated by significant (*α* = 0.05) main effects or interactions. A *p*-value <0.05 was considered to be significant.

## Supplementary material

As a service to our authors and readers, this journal provides supporting information supplied by the authors. Such materials are peer reviewed and may be re-organized for online delivery, but are not copy-edited or typeset. Technical support issues arising from supporting information (other than missing files) should be addressed to the authors.
