# Supplementary material for: MicroRNA expression profile and functional analysis reveal that miR-382 is a critical novel gene of alcohol addiction
Source: EMBO Mol Med. 2013 Jul 22;5(9):1402–14. doi: 10.1002/emmm.201201900 (PMC3799494; doi:10.1002/emmm.201201900)
Supplement: Supplementary file 1 [file emmm0005-1402-SD1.pdf]

Supplementary Information

Supplementary Information 1

Table SI: Comparison of membrane properties of NAc MSNs in acute brain slices of rats received NAc injection of saline or Ad-miR-382……………………………………………………...............2

Table SII: PCR Primer Sequences……………………………………………………………….2

Figure S1: Delivery of Ad-miR-382 to the rat NAc increases the expression of miR-382 in NAc, but not in the neighboring brain areas……………………………………………………….....3

Figure S1I: The successful up-regulation of miR-382 via Ad-miR-382 in NAc of rats at 7 days after drinking of alcohol…………………………………………………….................................3

Figure SIII: The successful modulation of DRD1 and DeltaFosB via Ad-miR-382 was verified by Western blot analysis at 7 days after drinking of alcohol ……………………………………....4

Table SI: Comparison of membrane properties of NAc MSNs in acute brain slices of rats received NAc injection of saline or Ad-miR-382.

Group	Saline or virus only	Ad miR-382
Number of neurons	29	16
Passive membrane properties		
RMP (mV)	-67.2 ± 3.0	-62.2 ± 1.8 (P=0.16)
R_{in} (MΩ)	344±67	533±133 (P=0.29)
Active membrane properties		
Current to generate AP (pA)	66.00 ± 17.13	87.50 ± 11.97 (P=0.20)
AP threshold (mV)	-31.7 ± 2.03	-35.5 ± 2.3 (P=0.27)
AP amplitude (mV)	62.9 ± 3.4	61.0 ± 3.2 (P=0.71)
½ AP duration (ms)	2.50 ± 0.18	2.3 ± 0.13 (P=0.65)
AHP amplitude (mV)	6.6 ± 1.4	5.5 ± 0.6 (P=0.40)

Note: The passive and active membrane properties of medium spiny NAc neurons in acute brain slice were measured by current clamp recordings from rat injecting either Ad-miR-382 or saline, or virus only. Since the results of saline injected and virus only were not difference they were pooled together. Values represent the mean ± SEM for the number of neurons indicated. RMP: resting membrane potential; AP: action potential; AHP: after-hyperpolarization. R_{in}, input resistance. The P values are obtained by t-test the difference between the two groups of neurons.

Table SI: PCR Primer Sequences

Primer Names	Sequences (5′->3′)
rat ΔFosB real-time forward primer	GAGGAAAAGGCAGAGCTGGA
rat ΔFosB real-time reverse primer	TGGGCCACCAGGACAAACT
rat DRD1 real-time forward primer	ACTTCGGCTCTGAAATCAGTTTGG
rat DRD1 real-time reverse primer	TCAACTCCTACCCTTCCTTTCTGG
mouse DRD1 real-time forward primer	GATGGCTCCTAACACTTCTACC
mouse DRD1 real-time reverse primer	GGCTGTGAGGATGCGAAAG
rat GAPDH real-time forward primer	AAGCTCACTGGCATGGCCTT
rat GAPDH real-time reverse primer	CGGCATGTCAGATCCACAAC

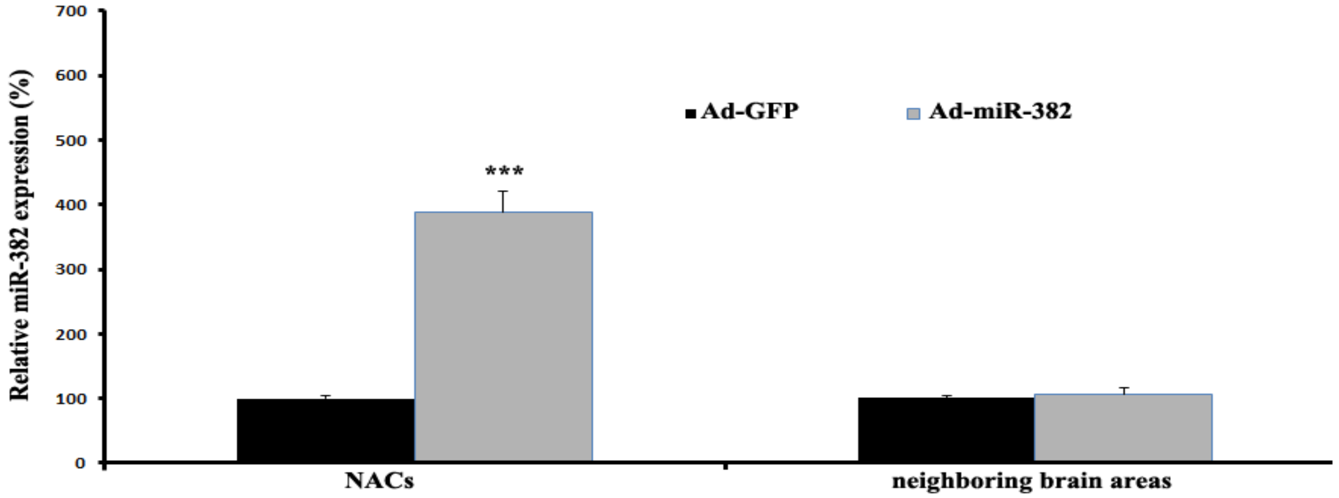

Figure S1. Delivery of Ad-miR-382 to the rat NAc increases the expression of miR-382 in NAc, but not in the neighboring brain areas. ****P*<0.0001, Student's t-test. Ad-miR-382 (4μl. 1×10^9 pfu/ml) or the control virus (4μl, Ad-GFP) was injected bilaterally into NAc core using a Kopf stereotaxic frame. Injections were performed with two 10 μl Hamilton syringe fitted with a micropipette. Four μl per side were injected into the NAc core at a rate of 0.1μl/min. At 5 days after injection, the rat NACs and neighboring brain areas (0.3 cm far from NAc) were isolated to determine the miR-382 levels by qRT-PCR. MiR-382 was increased via Ad-miR-382 in NACs (p= 1.22306E-5), but not in the neighboring brain areas**.** Values are mean ± SEM from 3 independent experiments (n=3), compared with that in Ad-GFP control.

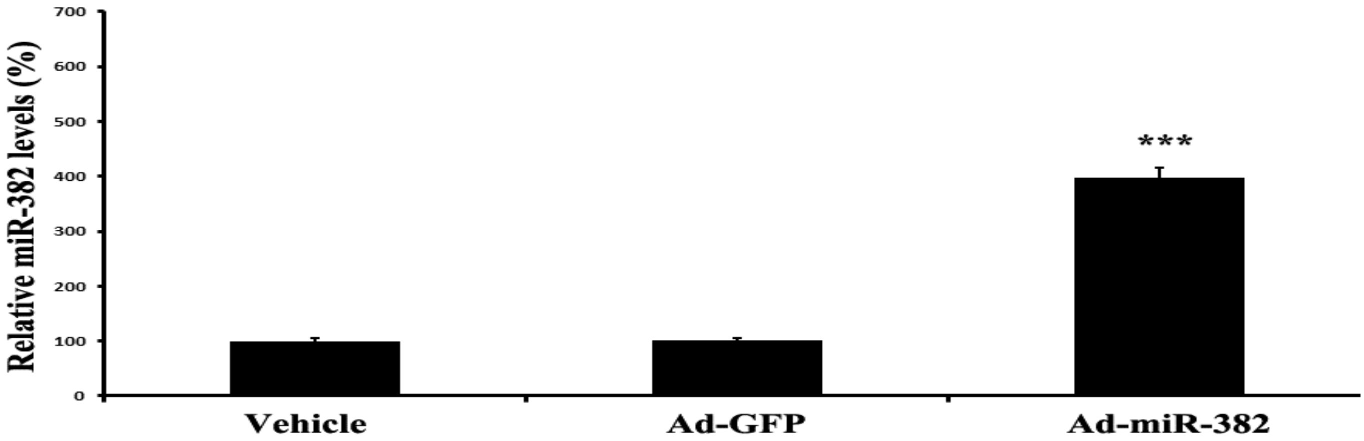

Figure SII. The successful upregulation of miR-382 via Ad-miR-382 in NAc of rats at 7 days after drinking of alcohol. ****P*<0.0001, Student's t-test. The animals under the intermittent access two-bottle choice drinking paradigm were randomly divided into three groups which received infusion of Ad-miR-382, control adenovirus Ad-GFP, or vehicle (saline) respectively into the NAc. Seven days later, NAc were isolated to determine the levels of miR-382 via qRT-PCR. MiR-382 was overexpressed via Ad-miR-382 (p=8.42076215208E-5). Values are mean ± SEM from 3 independent experiments (n=3), compared with that in Ad-GFP control.

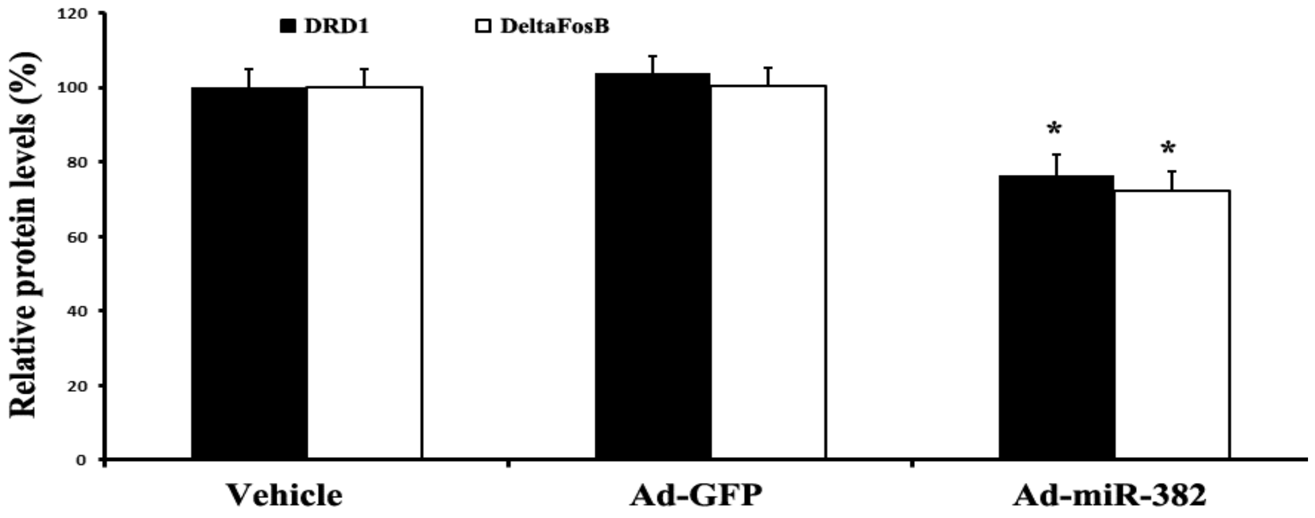

Figure S1II. The successful modulation of DRD1 and DeltaFosB via Ad-miR-382 in NAc of rats at 7 days after drinking of alcohol. **P*<0.05, Student's t-test. The animals under the intermittent access two-bottle choice drinking paradigm were randomly divided into three groups which received infusion of Ad-miR-382, control adenovirus Ad-GFP, or vehicle (saline) respectively into the NAc. Seven days later, NAc were isolated to determine the levels of DRD1 and DeltaFosB by Western blot analysis. Both DRD1 (p=0.01966) and DeltaFosB (p=0.01604) were downregulated by Ad-miR-382. Values are mean ± SEM from 3 independent experiments (n=3), compared with that in Ad-GFP control.
