# Supplementary material for: MicroRNA expression profile and functional analysis reveal that miR-382 is a critical novel gene of alcohol addiction
Source: EMBO Mol Med. 2013 Jul 22;5(9):1402–14. doi: 10.1002/emmm.201201900 (PMC3799494; doi:10.1002/emmm.201201900)
Supplement: Supplementary file 2 [file emmm0005-1402-SD2.pdf]

Source Data for Fig-2B

DRD1 → 49kD

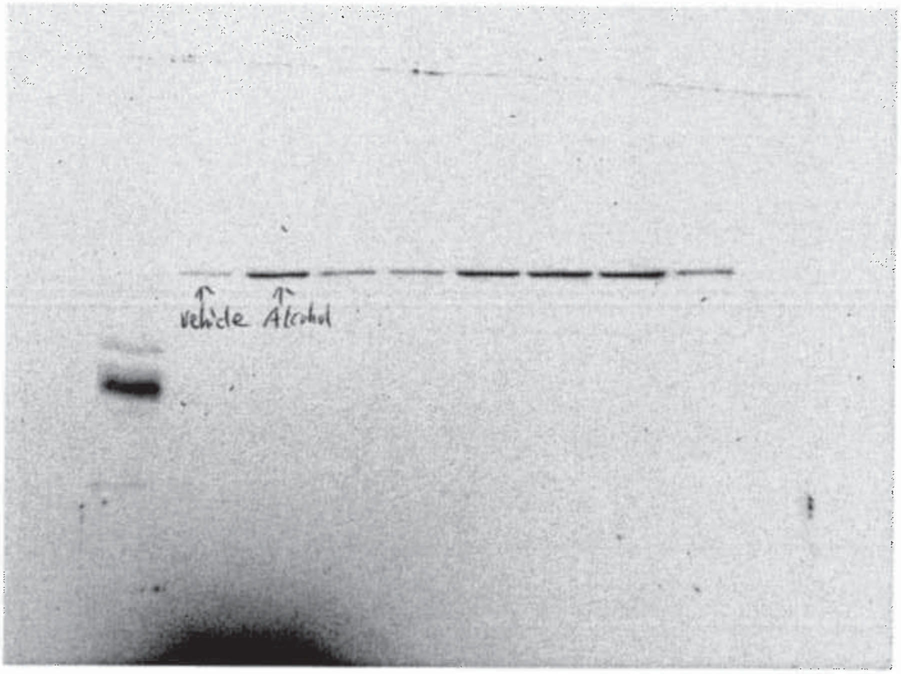

Source Data for Fig-2B

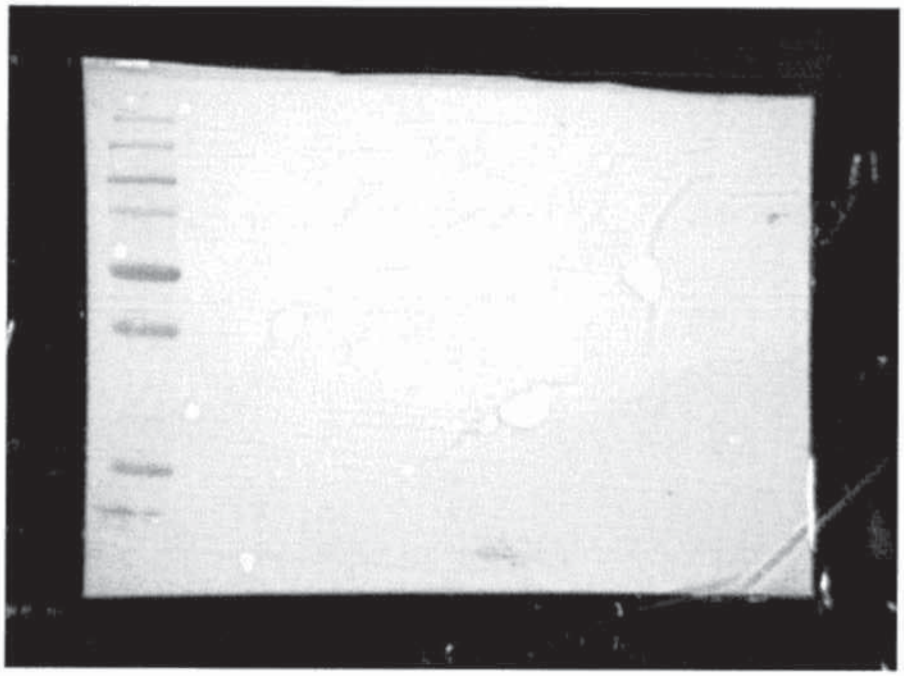

↑

Molecular Marker for DRD1-Study

Source Data for Fig-2B

Delta FosB -> 37kD

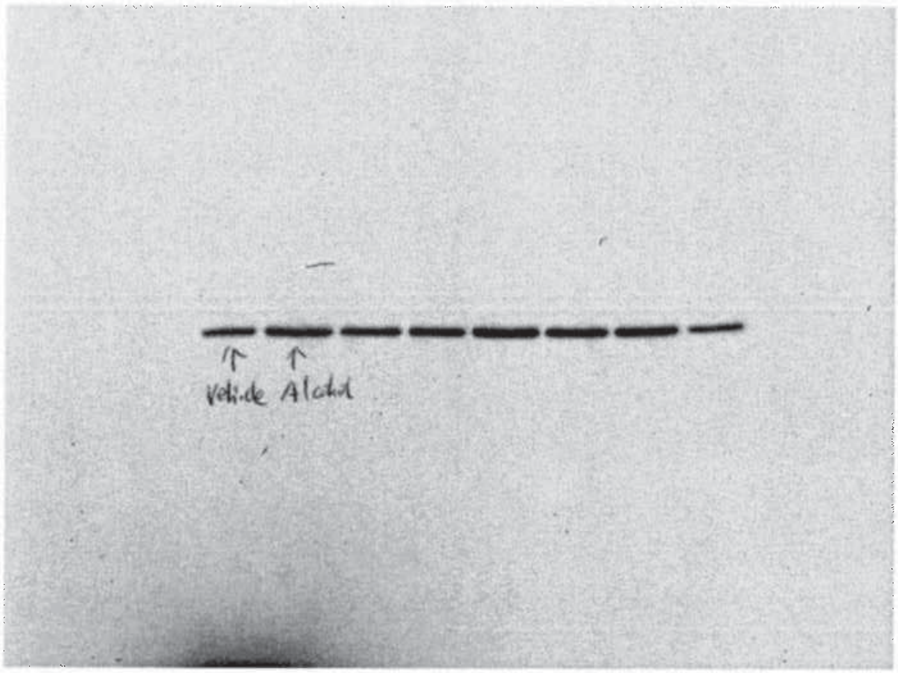

Source Data for Fig-2B

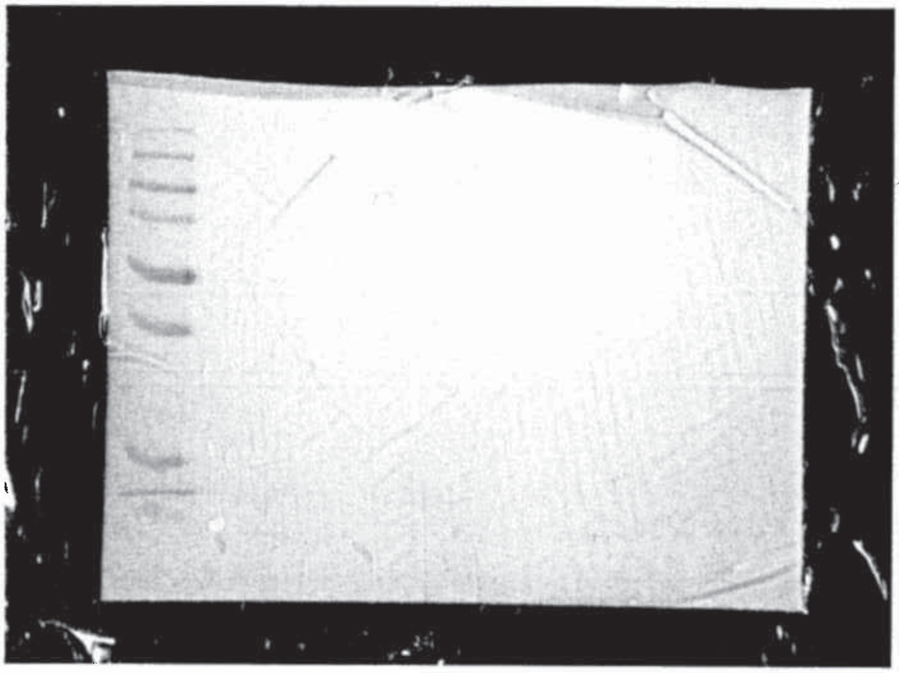

50KD →
37KD →
↑
Molecular marker for Delta FosB

Source Data for Fig-2B

GAPDH →

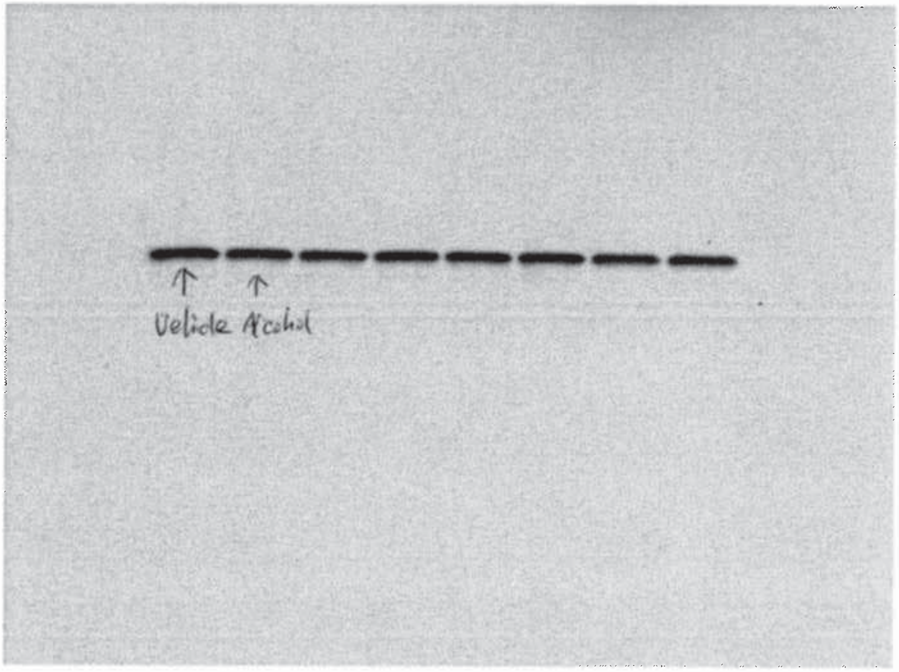

Source Data for Fig-2B

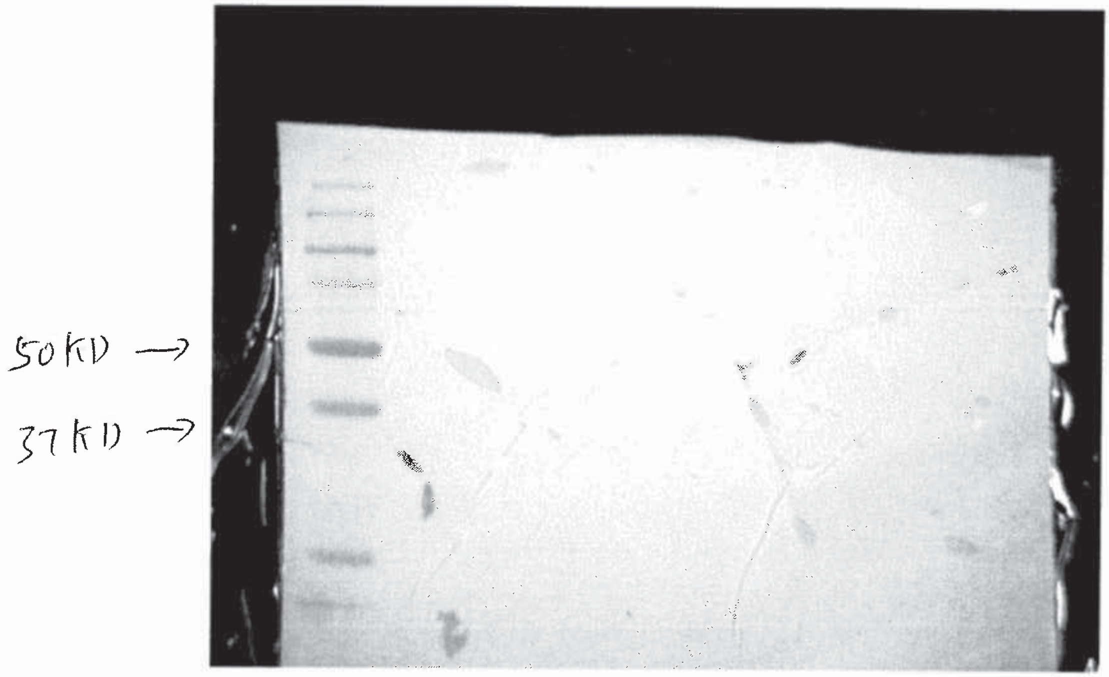

↑
Molecular Marker for GAPDH
