# Supplementary material for: MicroRNA expression profile and functional analysis reveal that miR-382 is a critical novel gene of alcohol addiction
Source: EMBO Mol Med. 2013 Jul 22;5(9):1402–14. doi: 10.1002/emmm.201201900 (PMC3799494; doi:10.1002/emmm.201201900)
Supplement: Supplementary file 3 [file emmm0005-1402-SD3.pdf]

Source Data for Fig-2F

DRD1 → 49 kD

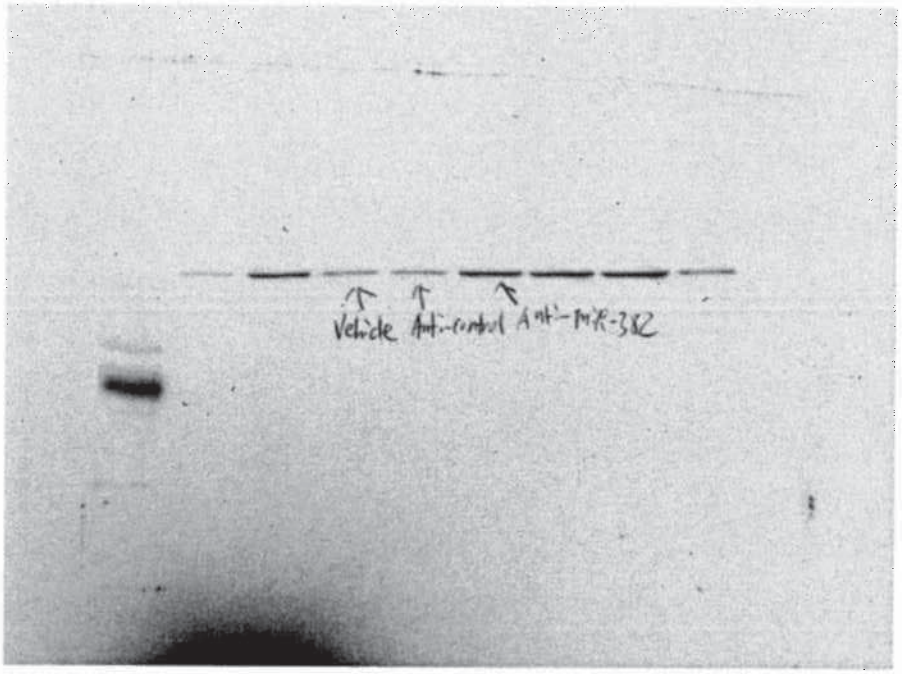

Source Data for Fig-2F

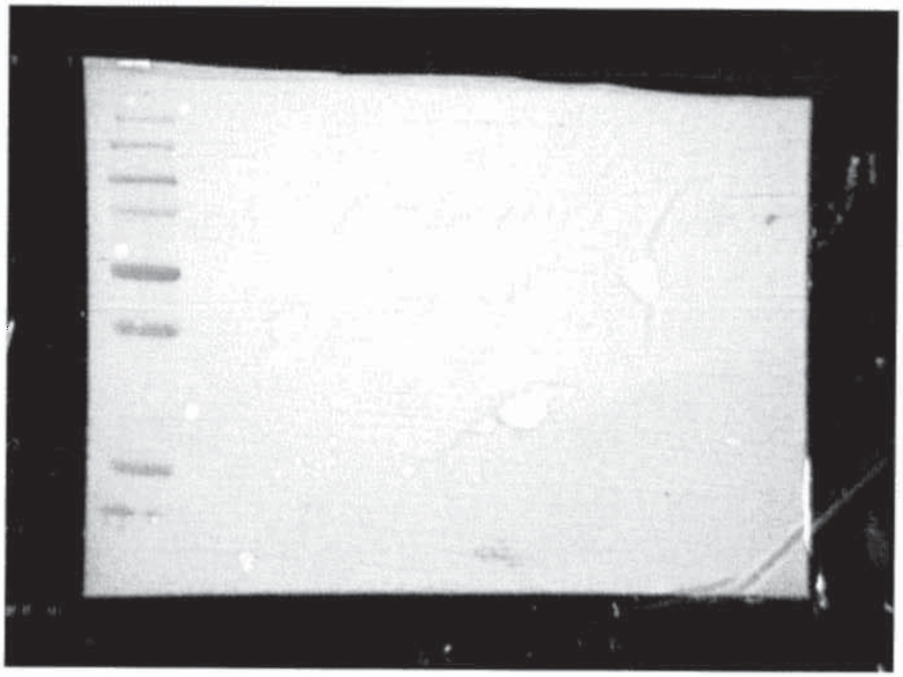


↑

Molecular Marker for DRD1- study

Source Data for Fig-2F

Delta FosB-37KD

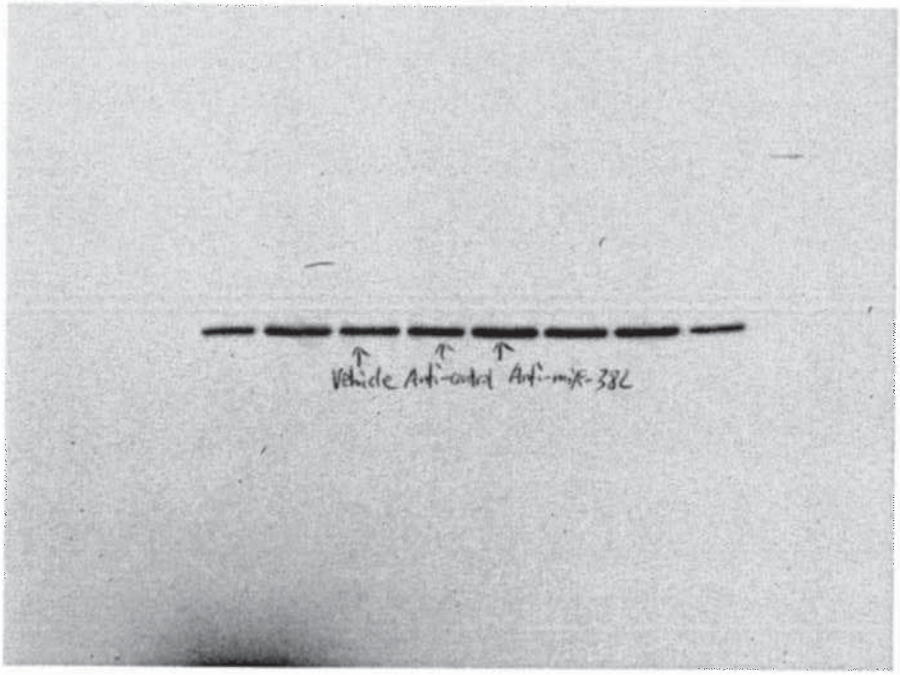

Source Data for Fig -2F

50KD →

37KD →

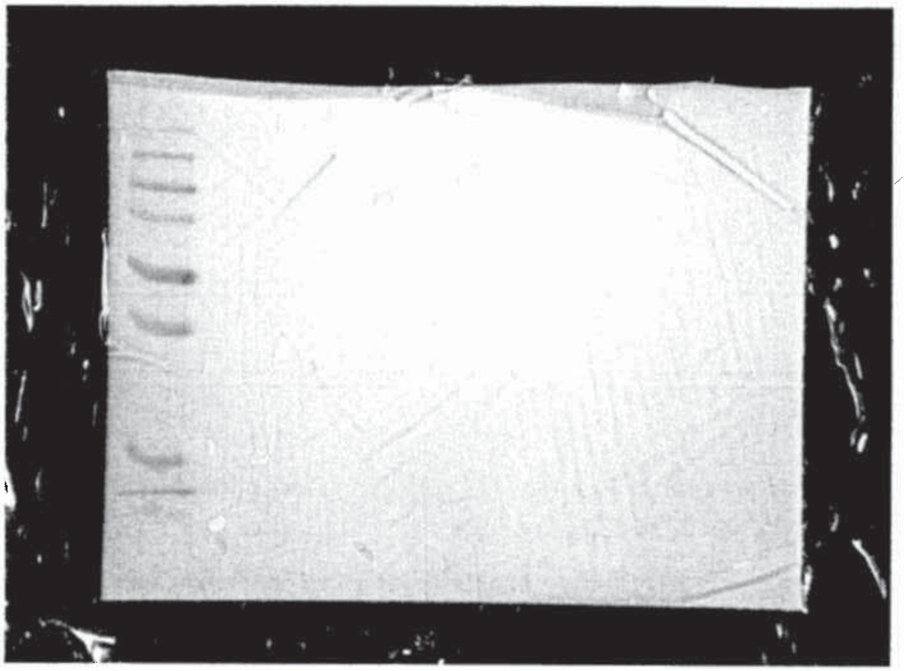

↑

Molecular Marker for Delta FosB

Source Data for Fig-2F

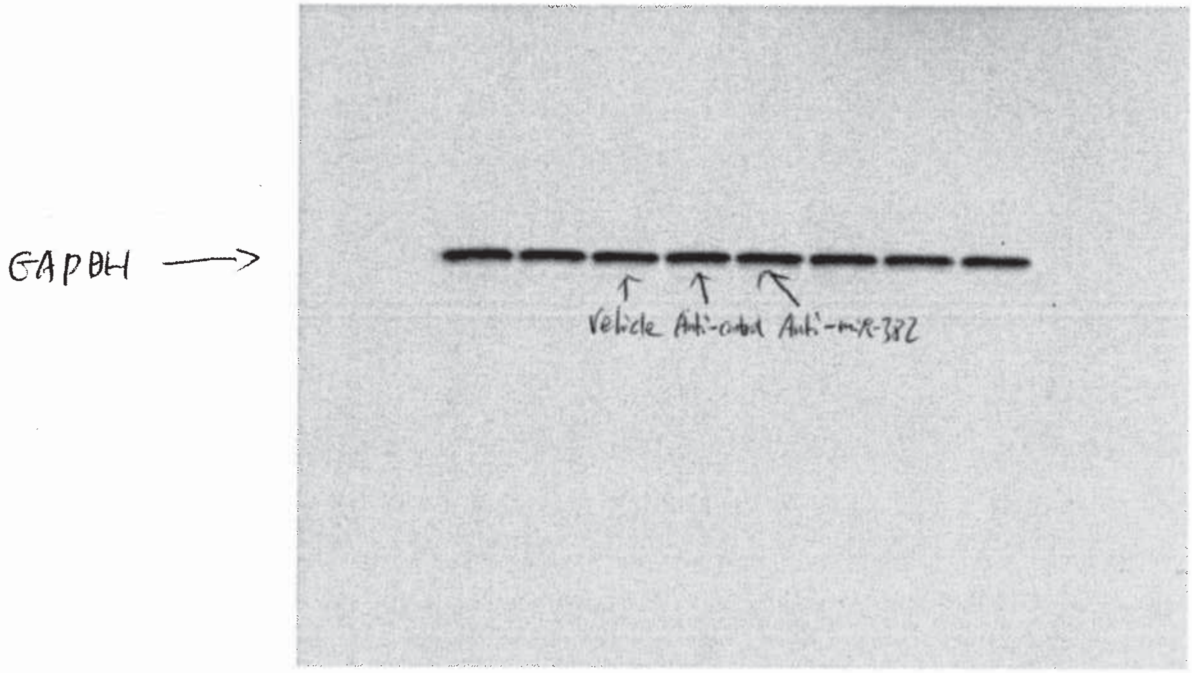

Source Data for Fig-2F

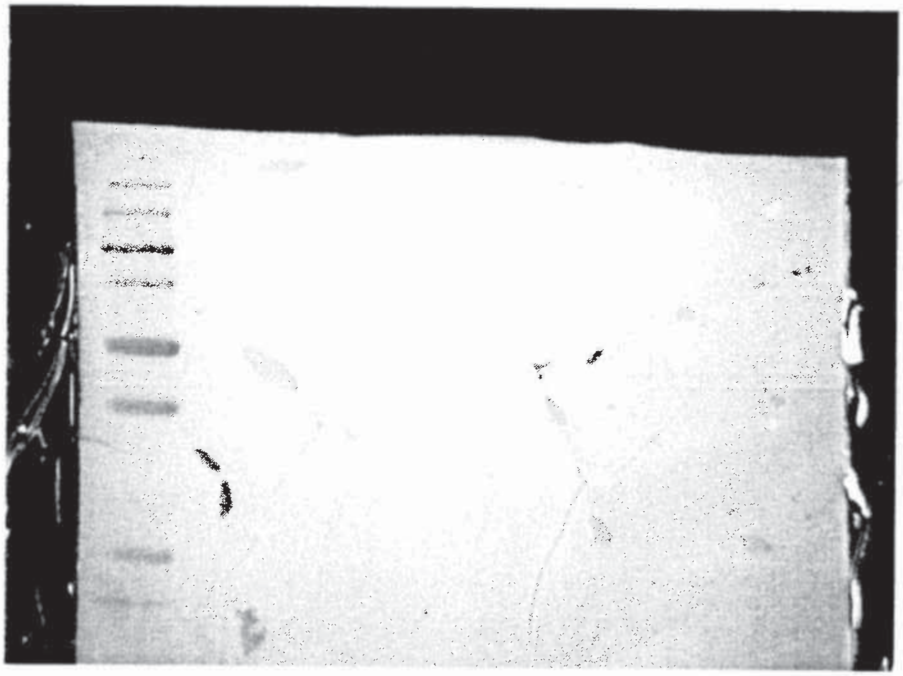


↑

Molecular Marker for GAPDH
