# Supplementary material for: MicroRNA expression profile and functional analysis reveal that miR-382 is a critical novel gene of alcohol addiction
Source: EMBO Mol Med. 2013 Jul 22;5(9):1402–14. doi: 10.1002/emmm.201201900 (PMC3799494; doi:10.1002/emmm.201201900)
Supplement: Supplementary file 4 [file emmm0005-1402-SD4.pdf]

Source Data for Fig-2H

DRD1 → 49KD

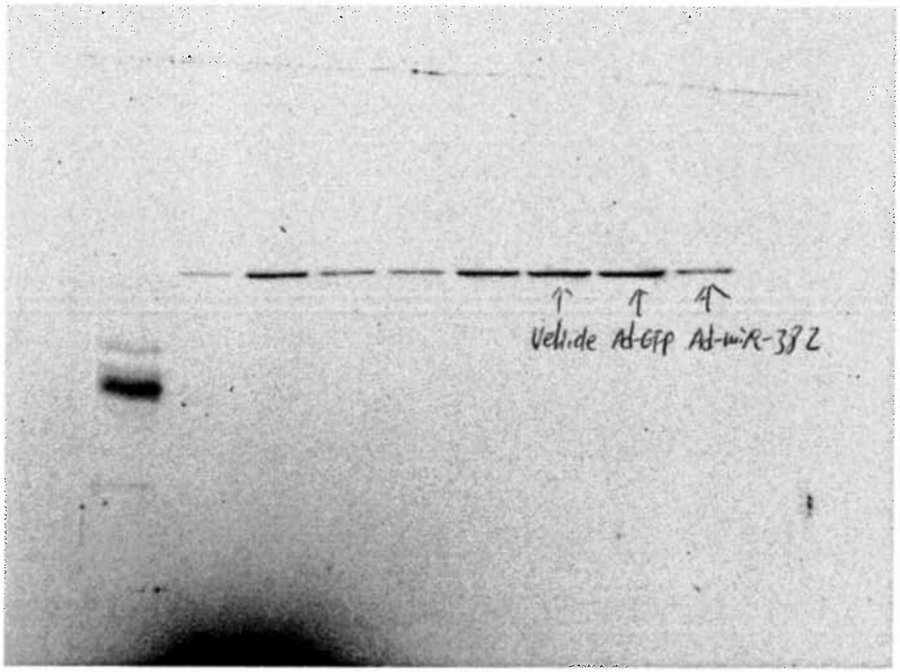

Source Data for Fig-2H

50 KD→
37 KD→

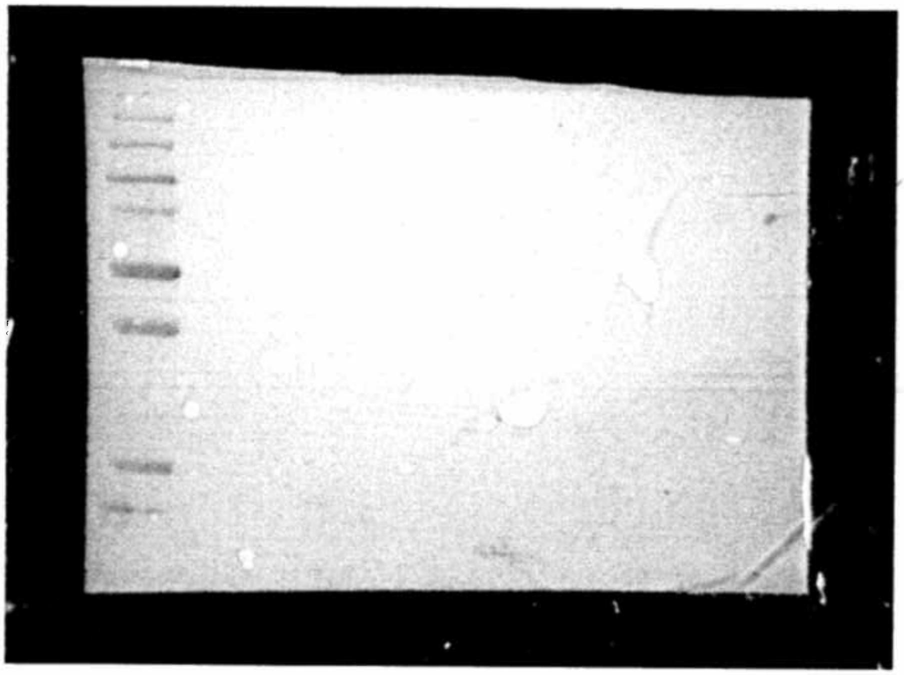

↑

Molecular Marker for DRD1- study

Source Data for Fig-2H

Delta-FosB → 37KD

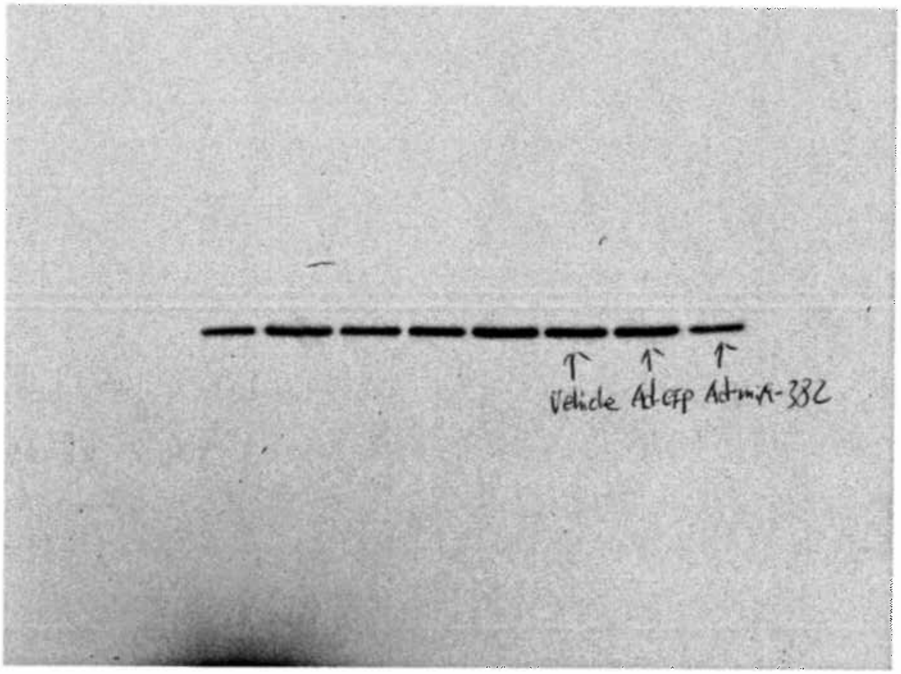

Source Data for Fig-2H

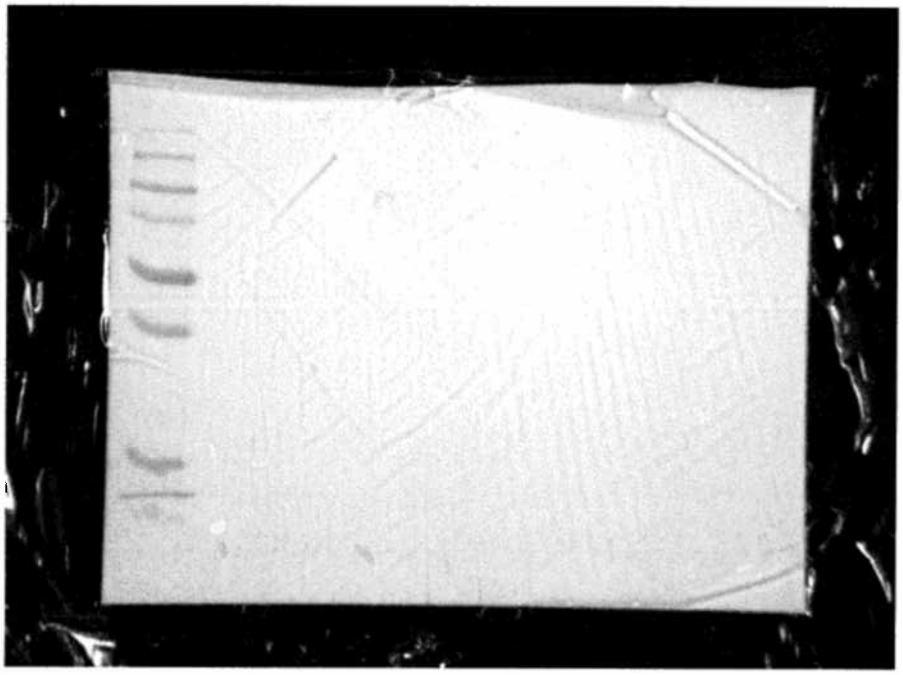

50KD →

37KD →

↑

Molecular Marker for Delta FosB

Source Data for Fig-2H

GAPDH →

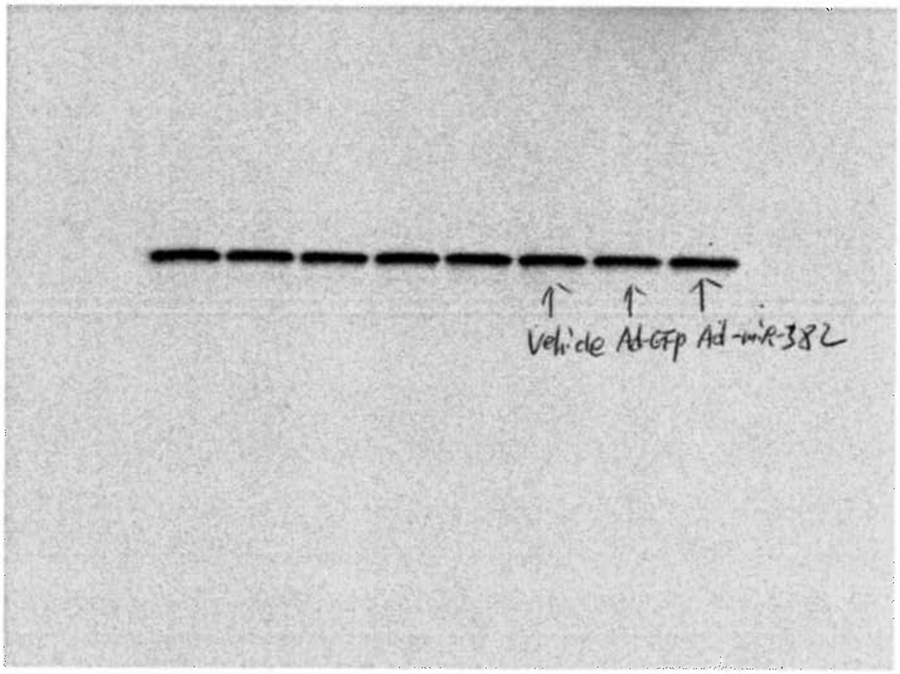

Source Data for Fig-2H

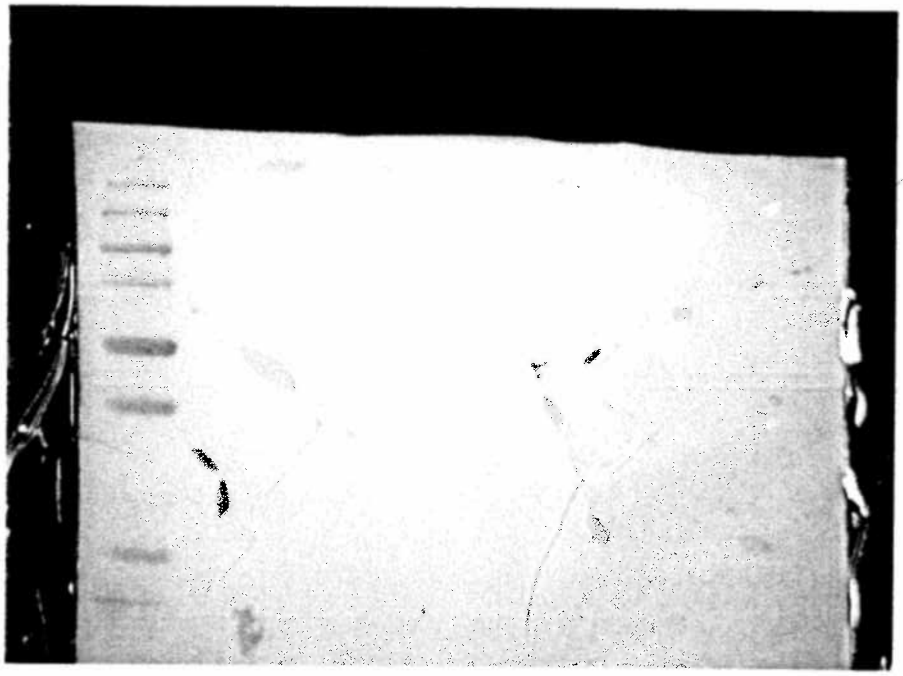

↑

Molecular Marker for GAPDH
