# Supplementary material for: MicroRNA expression profile and functional analysis reveal that miR-382 is a critical novel gene of alcohol addiction
Source: EMBO Mol Med. 2013 Jul 22;5(9):1402–14. doi: 10.1002/emmm.201201900 (PMC3799494; doi:10.1002/emmm.201201900)
Supplement: Supplementary file 5 [file emmm0005-1402-SD5.pdf]

Source Data for Fig-3E

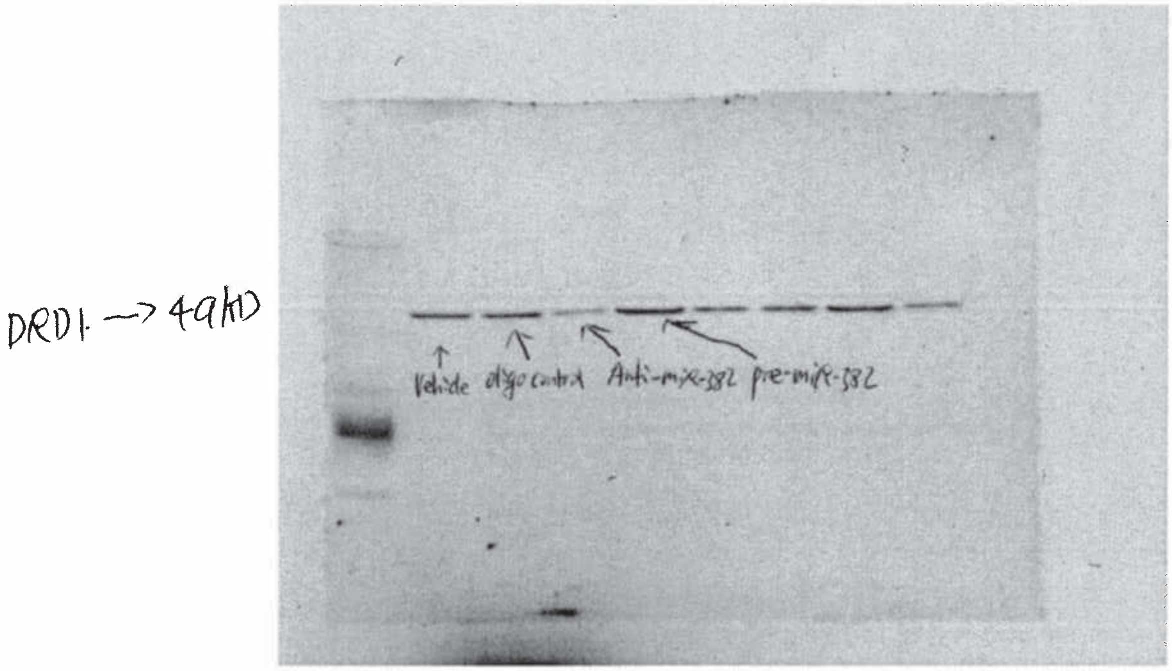

Source Data for Fig-3E

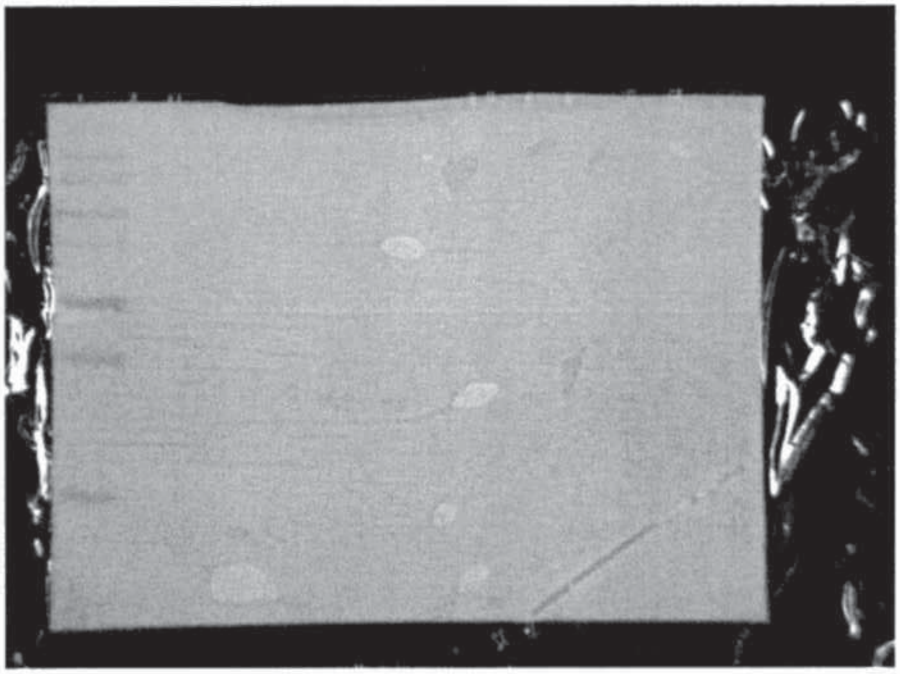

↑
Molecular Marker for DRD1 - study

Source Data for Fig-3E

Delta FosB→37KD

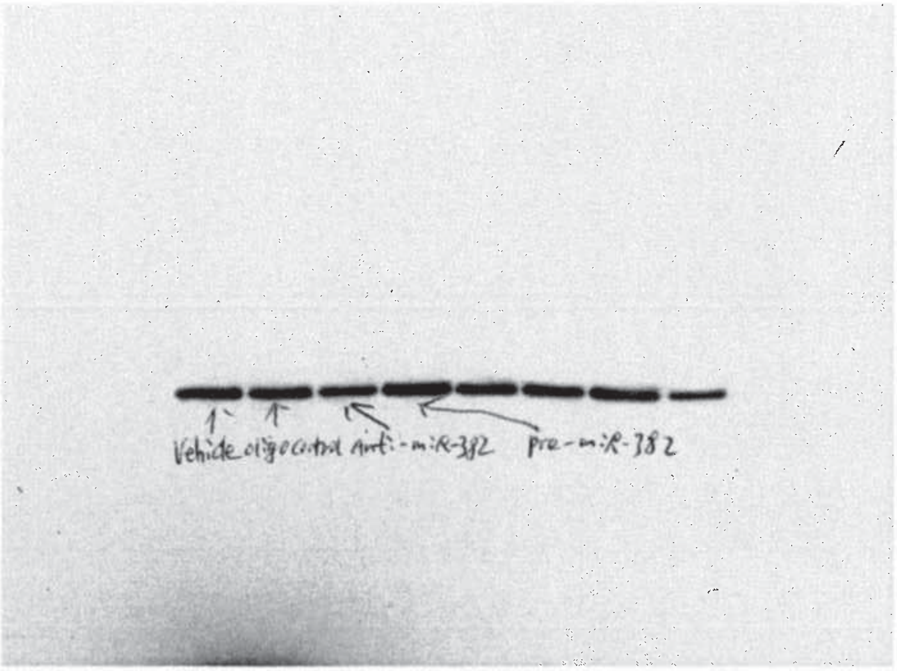

Source Data for Fig - 3E

50 KD →
37 KD →

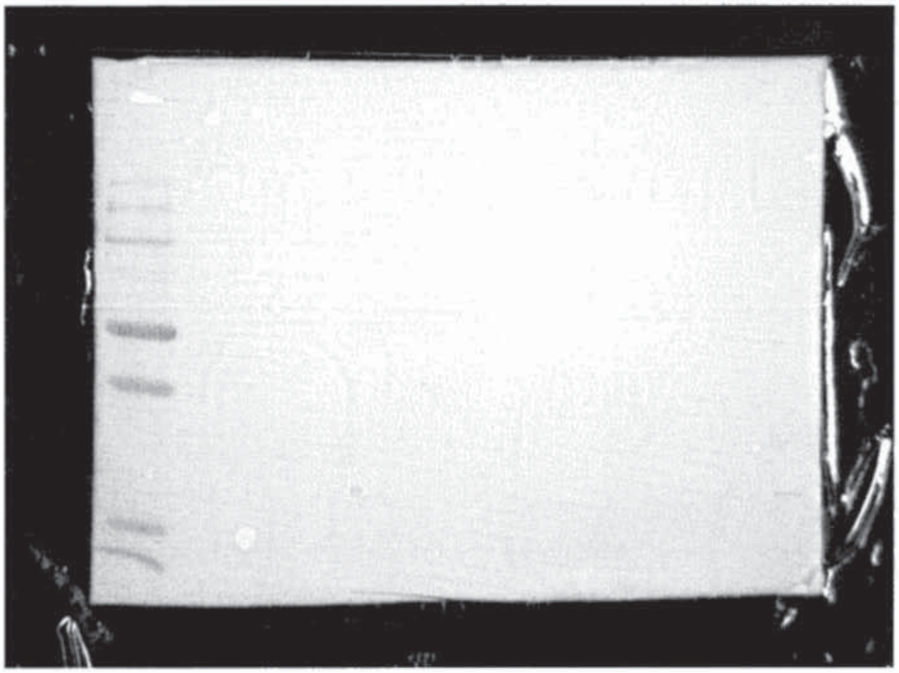

↑
Molecular Marker for Delta FosB

Source Date for Fig-3E

GAPDH

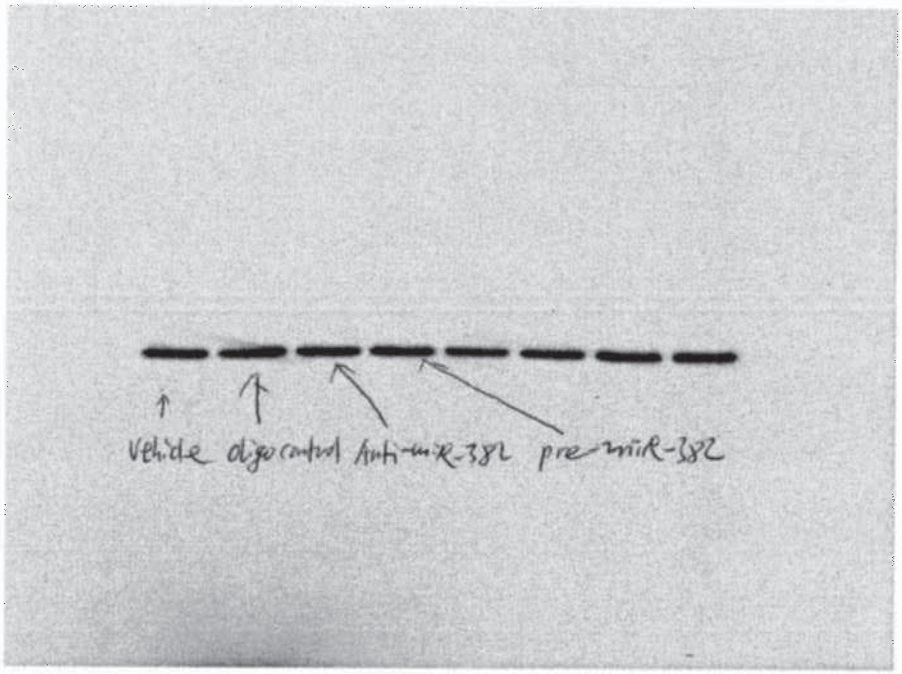

source Data for Fig - 3E

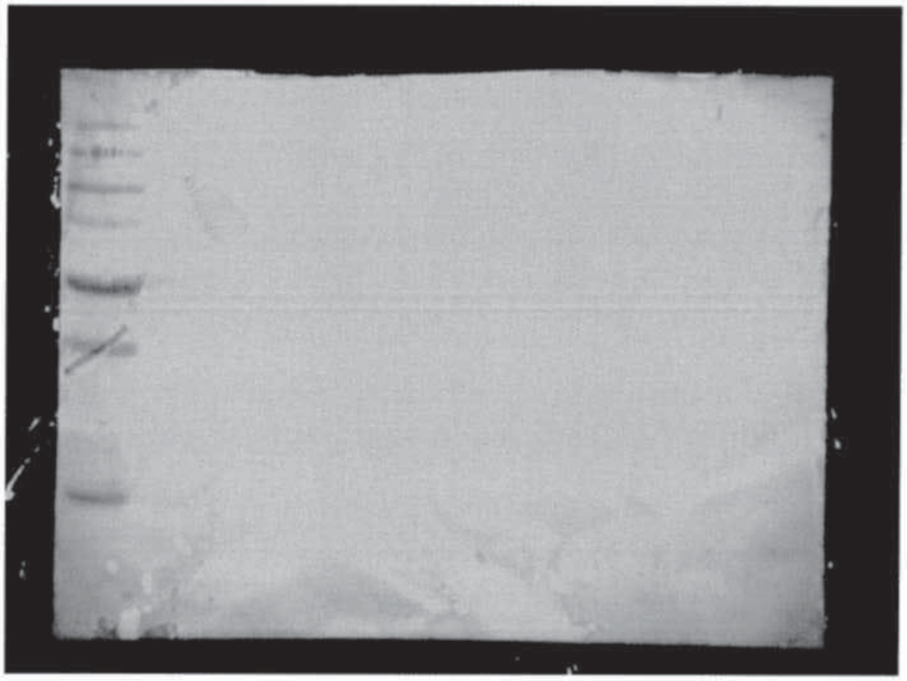

↑

Molecular marker for GAPDH
