# Supplementary material for: MicroRNA expression profile and functional analysis reveal that miR-382 is a critical novel gene of alcohol addiction
Source: EMBO Mol Med. 2013 Jul 22;5(9):1402–14. doi: 10.1002/emmm.201201900 (PMC3799494; doi:10.1002/emmm.201201900)
Supplement: Supplementary file 6 [file emmm0005-1402-SD6.pdf]

Source Data for Fig-4B

DRD1 → 49kD

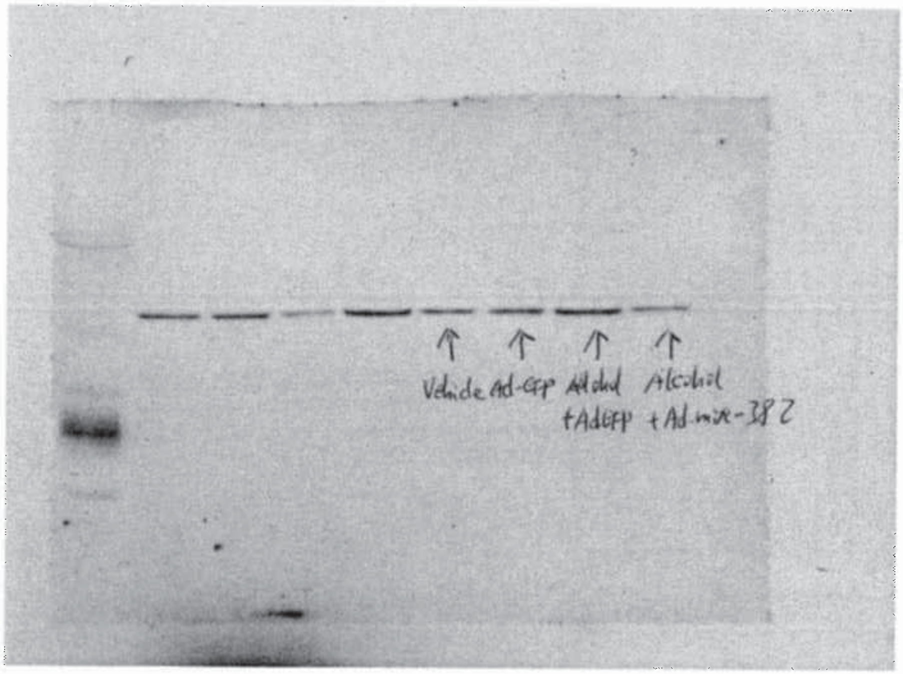

Source Data for Fig-4B

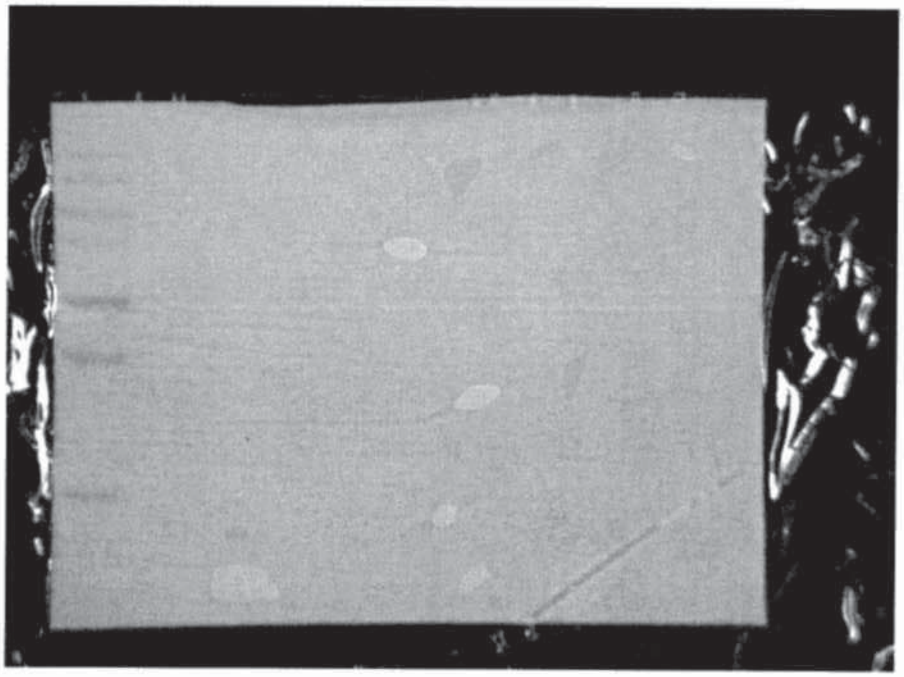

Molecular Marker for DRD1- Study

Source Data for Fig-4B

Delta FosB → 37KD

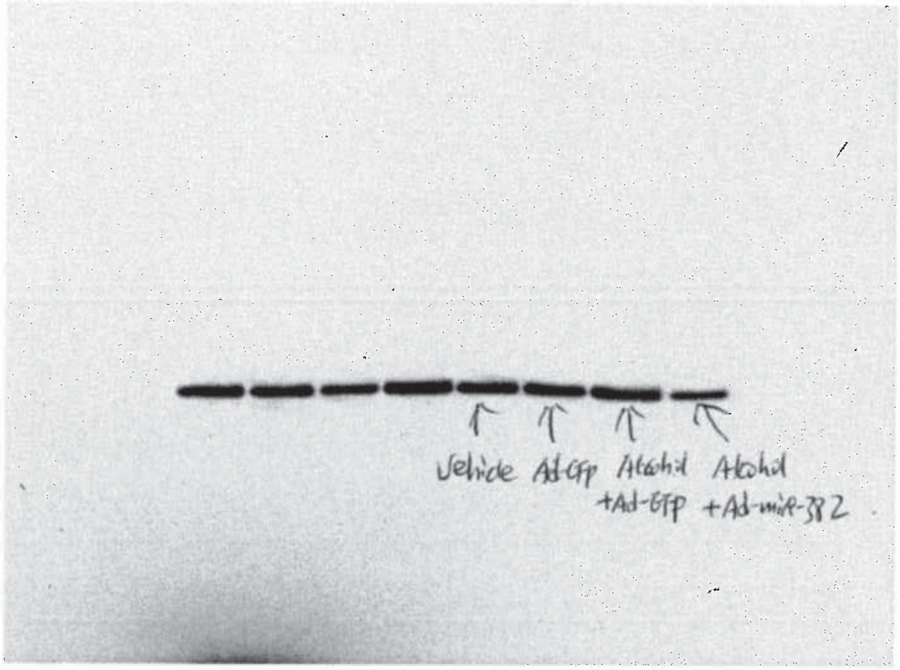

Source Data for Fig - 4B

50 KD →

37 KD →

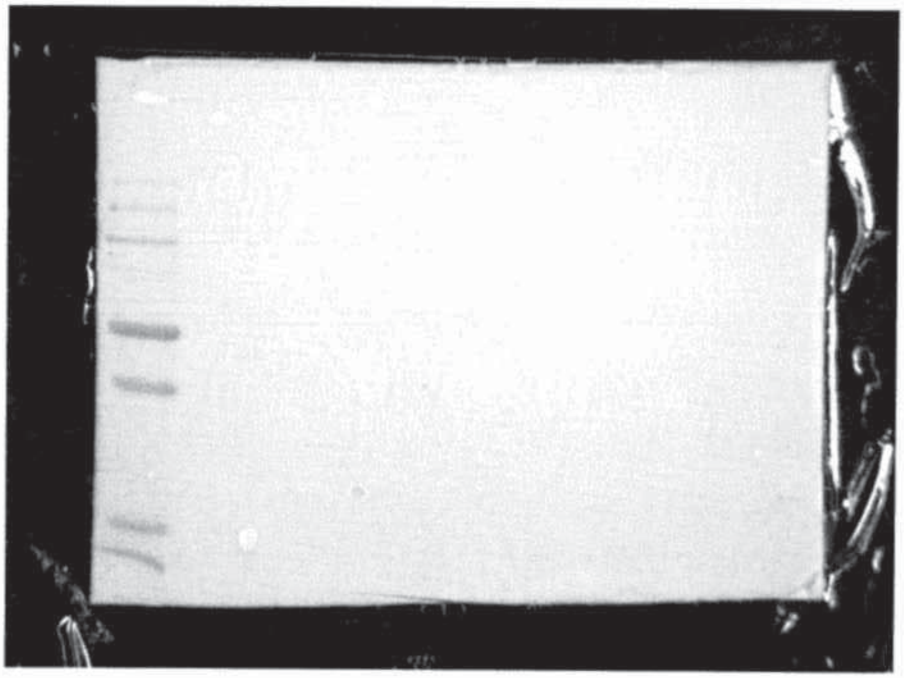

↑

Molecular Marker for Delta FosB

Source Data for Fig-4B

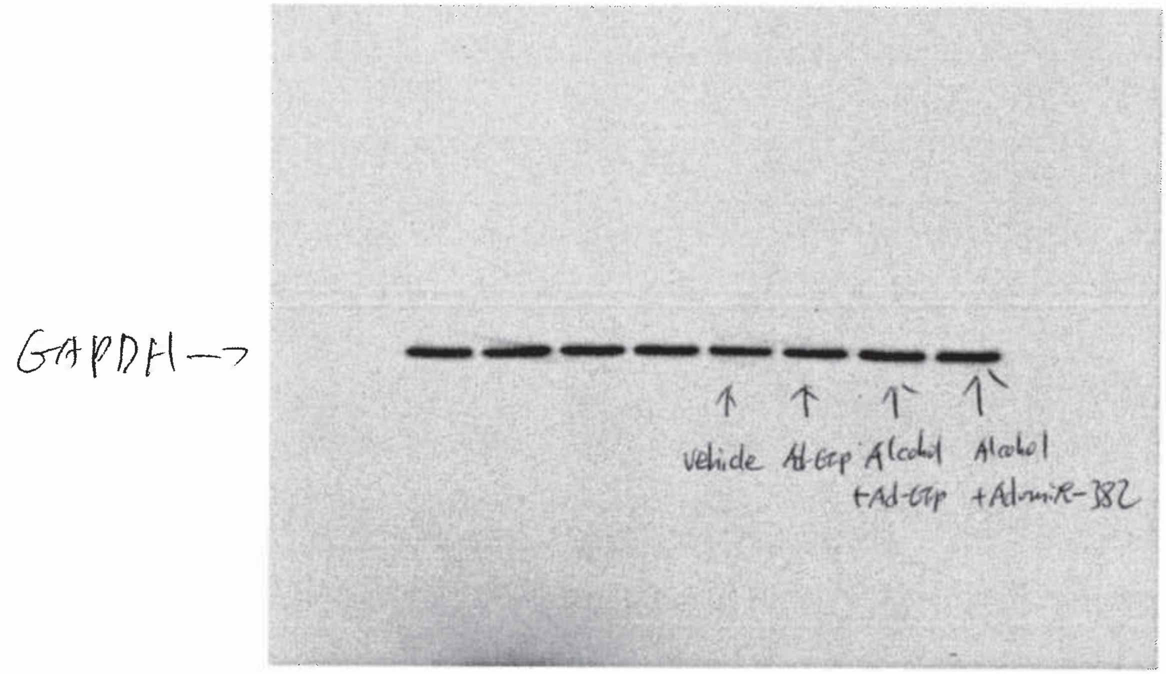

source Data for Fig-4B

50 kD –
37 kD →

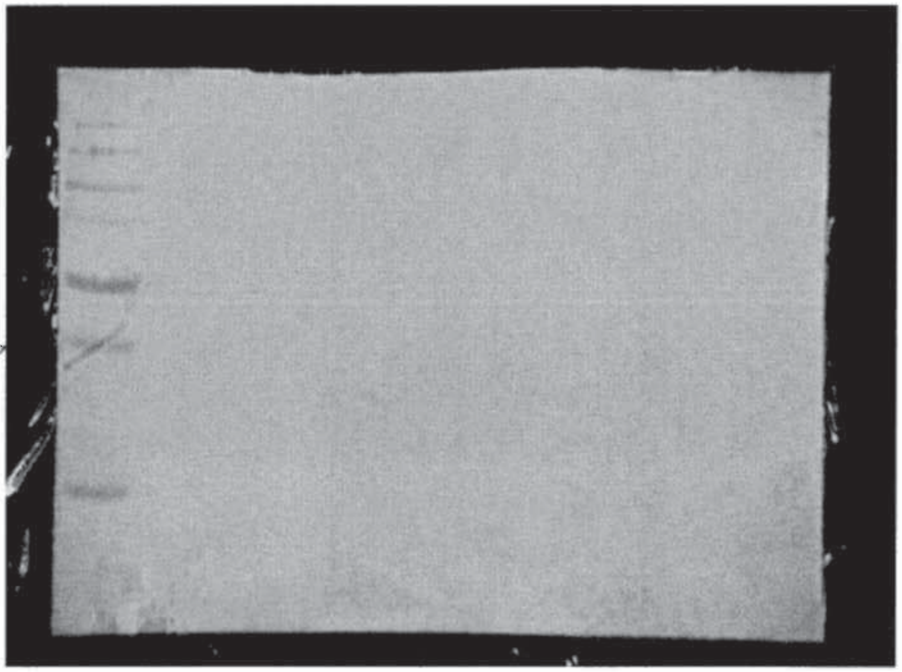

↑

Molecular marker for GAPDH
