# Supplementary material for: MicroRNA expression profile and functional analysis reveal that miR-382 is a critical novel gene of alcohol addiction
Source: EMBO Mol Med. 2013 Jul 22;5(9):1402–14. doi: 10.1002/emmm.201201900 (PMC3799494; doi:10.1002/emmm.201201900)
Supplement: Supplementary file 7 [file emmm0005-1402-SD7.pdf]

EMBO Molecular Medicine

MicroRNA Expression Profile and Functional Analysis Reveal that miR-382 is a Critical Novel Gene of Alcohol Addiction

Jingyuan Li, Jing Li, Xiaojun Liu, Shanshan Qin, Yanzhong Guan, Yuwei Liu, Yunhui Cheng, Xiuwen Chen, Wen Li, Shenming Wang, Ming Xiong, Eldo V. Kuzhikandathil, Jiang-Hong Ye, Chunxiang Zhang

Corresponding author: Chunxiang Zhang, Rush Medical College, Rush University

Review timeline:

	Submission date:	17 August 2012
	Accepted:	17 June 2013

Editor: Natascha Bushati / Céline Carret

Transaction Report:

No Peer Review Process File is available with this article, as the authors have chosen not to make the review process public in this case.
